# Predicting Prefecture-Level Well-Being Indicators in Japan Using Search Volumes in Internet Search Engines: Infodemiology Study

**DOI:** 10.2196/64555

**Published:** 2024-11-11

**Authors:** Myung Si Yang, Kazuya Taira

**Affiliations:** 1 Course of Advanced Nursing Sciences, Human Health Sciences, Faculty of Medicine, Kyoto University Kyoto Japan; 2 Human Health Sciences, Graduate School of Medicine, Kyoto University Kyoto Japan

**Keywords:** well-being, spatial indicator, infodemiology, search engine, public health, health policy, policy-making, Google, Japan

## Abstract

**Background:**

In recent years, the adoption of well-being indicators by national governments and international organizations has emerged as an important tool for evaluating state governance and societal progress. Traditionally, well-being has been gauged primarily through economic metrics such as gross domestic product, which fall short of capturing multifaceted well-being, including socioeconomic inequalities, life satisfaction, and health status. Current well-being indicators, including both subjective and objective measures, offer a broader evaluation but face challenges such as high survey costs and difficulties in evaluating at regional levels within countries. The emergence of web log data as an alternative source of well-being indicators offers the potential for more cost-effective, timely, and less biased assessments.

**Objective:**

This study aimed to develop a model using internet search data to predict well-being indicators at the regional level in Japan, providing policy makers with a more accessible and cost-effective tool for assessing public well-being and making informed decisions.

**Methods:**

This study used the Regional Well-Being Index (RWI) for Japan, which evaluates prefectural well-being across 47 prefectures for the years 2010, 2013, 2016, and 2019, as the outcome variable. The RWI includes a comprehensive approach integrating both subjective and objective indicators across 11 domains, including income, job, and life satisfaction. Predictor variables included *z* score–normalized relative search volume (RSV) data from Google Trends for words relevant to each domain. Unrelated words were excluded from the analysis to ensure relevance. The Elastic Net methodology was applied to predict RWI using RSVs, with α balancing ridge and lasso effects and λ regulating their strengths. The model was optimized by cross-validation, determining the best mix and strength of regularization parameters to minimize prediction error. Root mean square errors (RMSE) and coefficients of determination (*R*^2^) were used to assess the model’s predictive accuracy and fit.

**Results:**

An analysis of Google Trends data yielded 275 words related to the RWI domains, and RSVs were collected for 211 words after filtering out irrelevant terms. The mean search frequencies for these words during 2010, 2013, 2016, and 2019 ranged from −1.587 to 3.902, with SDs between 3.025 and 0.053. The best Elastic Net model (α=0.1, λ=0.906, RMSE=1.290, and *R*^2^=0.904) was built using 2010-2016 training data and 2-13 variables per domain. Applied to 2019 test data, it yielded an RMSE of 2.328 and *R*^2^ of 0.665.

**Conclusions:**

This study demonstrates the effectiveness of using internet search log data through the Elastic Net machine learning method to predict the RWI in Japanese prefectures with high accuracy, offering a rapid and cost-efficient alternative to traditional survey approaches. This study highlights the potential of this methodology to provide foundational data for evidence-based policy making aimed at enhancing well-being.

## Introduction

### Conceptualizing Well-Being

Well-being is a complex and multifaceted concept for which there is “no consensus around a single definition of well-being”, as acknowledged by the US Centers for Disease Control and Prevention, but generally, well-being refers to “judging life positively,” “feeling good,” and the experience of good physical health [1]. It also includes dimensions such as physical health, emotional health, economic circumstances, life satisfaction, and engaging activities and works [2]. In the early 21st century, numerous scholars suggested that well-being should be quantified to create indicators that could guide national public policies. Consequently, many scales have been proposed [2-5]. However, the absence of a universally accepted definition of well-being has made it challenging to establish a single, comprehensive measure [6]. Current measures often fall into categories such as “life evaluation,” “hedonic well-being,” and “eudaimonic well-being,” each capturing different aspects of the well-being spectrum [7]. Yet, because these dimensions are deeply interconnected, scales focused on specific aspects may not fully capture the overall well-being of individuals.

### The Need for a Comprehensive Well-Being Indicator

Historically, well-being has been measured primarily by economic indicators such as gross domestic product (GDP). However, a prior study has suggested that economic indicators are “no longer a complete approximation of how well a nation is doing” [4]. GDP is only one aspect of well-being and does not capture socioeconomic inequalities, life satisfaction, or health status [8]. For the nation as a whole, previous research suggests that as the GDP or economic activity increases, the standard of living of the population improves [9]; however, in a well-developed society, there may be some citizens who sacrifice their well-being for economic efficiency. In fact, economic growth can lead to improved living conditions in some areas, but an examination of the United States revealed that despite a 3-fold GDP increase in the past 50 years, life satisfaction levels have not risen [3], accompanied by rising depression and anxiety rates [10]. Therefore, GDP growth is not equal to improved well-being, and more comprehensive well-being indicators that include multiple factors such as health, work, and social connections need to be used in policy evaluation. However, it is not proposed to completely replace traditional economic indicators, but to add new indicators of well-being and look at the relationship between both the economy and well-being [11].

### Well-Being Indicators Currently Used for Policy Evaluation and Their Challenges

Several countries and international organizations use a variety of different well-being indicators, which are a mix of subjective and objective indicators. Organizations such as the Organization for Economic Co-operation and Development (OECD) and United Nations Development Program, as well as countries including New Zealand, the United Kingdom, France, and Italy, use objective well-being metrics encompassing health (eg, lifespan), job opportunities (eg, employment rate), environmental conditions (eg, greenhouse gas emissions), safety (eg, crime rate), and governance (eg, voter turnout) [12-18]. Subjective indicators involve self-evaluations of individuals of their lives, as exemplified by tools such as the Satisfaction With Life Scale [19] and the Subjective Happiness Scale [20]. In practical policy implementation, dashboards that visualize data for both objective and subjective indicators have been devised to holistically assess social well-being. In countries like the United Kingdom and New Zealand, unique well-being indices have been developed to gauge citizen welfare and guide policy and fiscal decisions [15-18].

In addition, some international organizations are involved in the development of well-being indicators and are publishing reports on international comparisons. Typical examples include the Better Life Index (BLI) of the OECD [12], the World Happiness Report of the United Nations [13], and the Human Development Index of the United Nations Development Program [14]. Moreover, while many well-being indicators target national units, there are initiatives to establish metrics for more localized regional assessments within countries [21], illustrating diverse methodologies to evaluate comprehensive well-being.

These preexisting well-being indicators have some limitations and challenges. Conducting large-scale epidemiological surveys, which are necessary for both objective and subjective indicators, incurs significant human, temporal, and financial costs. In several resource-limited nations, conducting surveys can be challenging, or individuals experiencing severe well-being deficiencies are often more prone to nonresponse, thereby hindering the assessment of their well-being. In addition, survey items rely on multiple government surveys, and survey years often vary and can only be evaluated once every few years. It is also difficult to standardize the items surveyed across all countries, making international comparisons difficult. Therefore, a reasonable indicator that is comparable to other regions can be obtained, and evaluated at the right time for policy evaluation is very useful for policymakers.

### Web Log Data as a Policy Indicator

With the spread of the internet, methods for using web log data to predict statistics for policy evaluation have been reported. Statistics related to well-being indicators have also been associated with web log data, including health [22], job availability [23], environmental quality [24], safety [25], governance [26], and subjective elements, such as emotional well-being [27] and life satisfaction [28]. The web log data used in the previous study varied and included search volume logs from internet search engines, such as Google and Yahoo! Search, as well as log data from social networking services (SNSs), including X (Twitter), Facebook, and Instagram. This study used search volume log data from the search engine Google, which can be collected from Google Trends [29]. Google is one of the major search engines used in 195 countries around the world, making it easy to ensure reproducibility in other countries. Another advantage of social networking tools is that there is less bias in demographic information such as user age, gender, and race. While information posted on SNSs is often directed toward society and others and may contain only overly idealistic information, search behavior on search engines is an individual’s internal process and might be able to reduce the confirmation bias that amplifies the information that people find favorable.

### Objective

This study aimed to develop a model that predicts comprehensive well-being indicators via search volume log data from internet search engines. This approach seeks to bypass the need for large-scale statistical surveys, thereby reducing budgetary and human resource requirements. In other words, it enables policymakers to assess the well-being of the public at low cost and at the right time, thereby facilitating more effective policy decisions.

## Methods

### Regional Well-Being Index as an Outcome

The Regional Well-Being Index (RWI) for Japan [30], structured based on the BLI methodology of the OECD, was used as the outcome variable. The RWI, like the BLI, consisted of 11 domains: “Income,” “Jobs,” “Housing,” “Health,” “Work-Life balance,” “Education,” “Community,” “Civic Engagement,” “Environment,” “Safety,” and “Life Satisfaction.” This index focuses on integrating both subjective and objective indicators to comprehensively evaluate well-being.

The “Regional Well-Being” of the OECD [21] provides detailed scores for well-being at subnational regions at smaller geographical scales than the national level. However, the administrative divisions that make policymaking do not coincide with these subnational regions in several countries. For instance, the OECD’s regional well-being for Japan is presented at a relatively macroscopic level, segmenting the nation into 10 regions, including Tohoku and Kansai, each encompassing several prefectures. This level of aggregation differs from the levels at which policy decisions are operationalized, typically at the prefectural and municipal levels. To use well-being indicators more efficiently, it is important to calculate them for each administrative level where policy evaluation and decision-making are conducted. Therefore, this study adopted the RWI, a comprehensive well-being indicator at the prefectural level in Japan, based on the BLI methodology, as its outcome.

Yang and Taira [30] provided domain-specific scores and an integrated RWI (IRWI) that aggregated all domains by prefecture. These RWIs assessed the well-being of all 47 Japanese prefectures for 2010, 2013, 2016, and 2019, affirming their reliability and validity relative to the BLI and the existing well-being index [30]. Due to the data unavailability of certain indicators constituting the RWI, the 2019 data remain the most recent. Subsequent updates have been delayed due to factors such as the COVID-19 pandemic and prolonged data aggregation processes.

### Internet Search Log Data as Predictor Variables

The relative search volume (RSV) in the internet search engine Google was used as the predictor variable and was obtained from the Google Trends website. Google Trends enables the tracking of temporal variations in the popularity of specific search words on Google and YouTube while capturing regional search dynamics and related words. Google Trends was used to collect related words in addition to the RSVs for the main words. The “Related keywords” feature of Google Trends offers other words that are frequently searched in relation to a selected word. “Related keywords” include “Top searches” and “Rising searches,” where “Top searches” are the words most frequently searched within the same session as the selected word, and “Rising searches” are the words whose search frequency has increased the most significantly over a specific period. Google Trends collects up to 25 related words.

In Google Trends, RSVs are normalized on a 0-100 scale, where the word with the highest search volume in a specific period scores 100, and the frequencies of other words are adjusted accordingly. Moreover, Google Trends facilitates the comparison of search frequencies for words across different states or prefectures within a country, normalizing data to the area with the highest frequency of a word score of 100, whereas others are rated in comparison. For instance, a search frequency score of 50 for a word in one region suggests that its search volume is half of that of the highest-ranked region.

**Figure 1:**
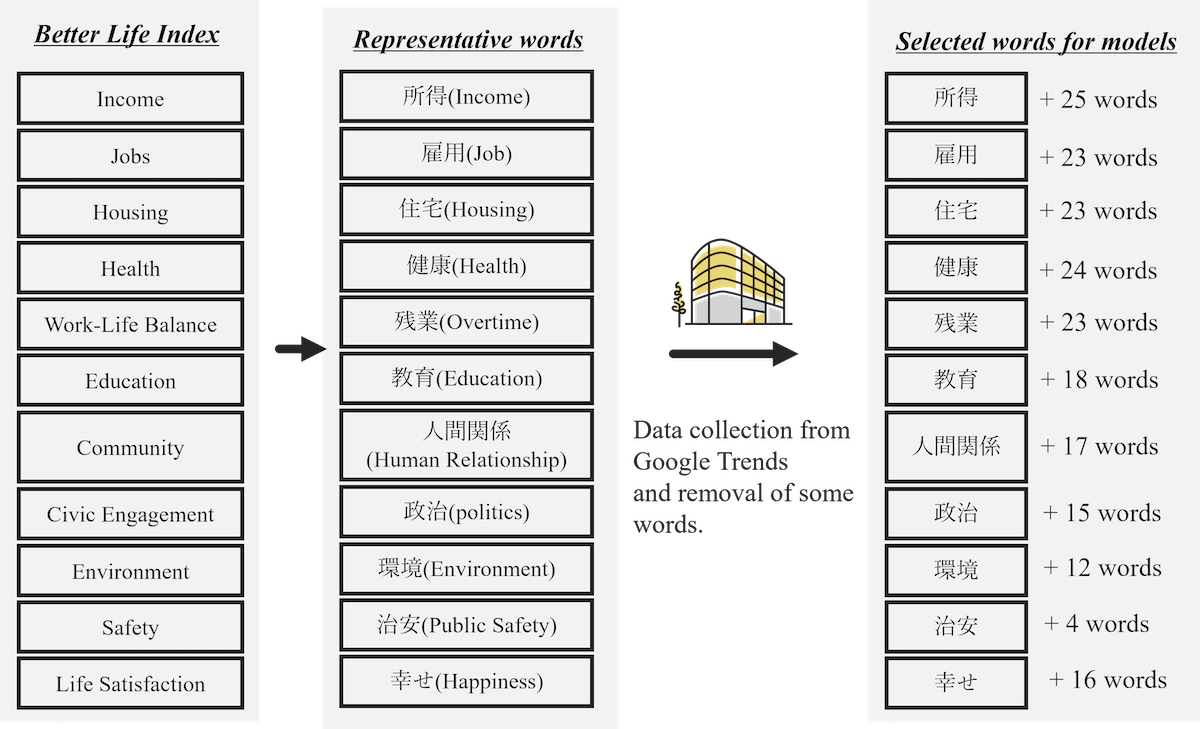
Creation method for the word set. “Representative words” provide the English translations of the original Japanese words in parentheses.

First, in the procedure for obtaining RSVs, following the methodology established in prior research that developed the RWI framework, representative words for each domain were selected based on their relevance to the specific domain, ensuring alignment with their Japanese translations (Figure 1). After selecting the main word for each domain, related search words were collected using Google Trends, which can gather up to 25 related words for any given word. The collection period spanned between January 1, 2010, and December 31, 2019, aligned with the RWI measurement years, and RSVs of related search words during this period were collected. In addition, we collected RSVs for web searches within Japan, without limiting them to any specific category but including all categories. Data collection was conducted without using quotation marks around the search words. Since searching multiple words simultaneously would result in normalization with the word having the highest search volume set to 100, we collected RSVs for each term individually.

To extract the words for the predictive model, those that included specific regions, companies, or personal names were excluded. Additionally, 2 authors, YM and TK, double-checked and deleted words irrelevant to each domain. For example, words such as “福祉 住 環境” (welfare living environment) relating to the environment of an individual’s residence were judged inappropriate and removed, since the original “environment” domain contains the words referring to the “natural environment.” In the Community domain, words like “人間関係” (human relations) and “人間 関係,” differing only in spacing, were identified to represent the same concept. Since the search trends were similar (Multimedia Appendix 1) and it was considered that searchers had the same intent, only “人間関係” was included.

Finally, we collected the RSVs for all main and related words by prefecture. Google Trends allows for the comparison of search frequencies of specific words across states or prefectures within a selected country using its “Interest by Subregion” function. The function provides a country map shaded according to the term’s popularity. The color intensity represents the percentage of searches for the leading search term in a particular region. Search term popularity is relative to the total number of Google searches performed at a specific time, in a specific location. Data were collected for each keyword by setting specific time frames for the years 2010, 2013, 2016, and 2019 (eg, January 1, 2010, to December 31, 2010). In Google Trends, extremely low search frequencies are sometimes indicated as “less than 1” instead of “0,” such cases were treated as “0” in this study. The normalization process for RSVs was conducted for the data of each domain within each year, standardizing the annual prefecture-specific data. This means, for example, that the prefecture-specific data in the “Income” domain for the year 2010 was standardized to ensure comparability across prefectures.

### Statistical Analysis

A descriptive statistical analysis of the RWIs and RSVs was conducted for all words. We calculated the mean and SD of the RSVs for representative words of each domain for the years 2010, 2013, 2016, and 2019 by the prefecture to explore temporal variations in search behaviors across different regions. We also calculated Pearson partial correlation coefficients by adjusting the data year and population of prefectures [31] to evaluate the association between the RWI scores and the RSV of each keyword.

As a supplementary analysis, we conducted a spatial evaluation of IRWI scores to assess the geographical interrelationships of well-being across regions. Well-being may be influenced by regional cultural characteristics, leading to similarities in IRWI scores among neighboring areas. Understanding these spatial relationships can provide insights for considering regional collaboration in policy interventions.

We first applied Global Moran *I* [32] to assess the overall spatial autocorrelation of IRWI scores across Japan, identifying whether scores were clustered or dispersed on a national level. A significant positive Global Moran *I* indicates the clustering of similar values, while a negative value suggests the dispersion of dissimilar values. Additionally, Local Moran *I* [33] was used to analyze local similarities and differences between regions and their neighbors, allowing us to identify clusters of high or low scores. This analysis highlights regional disparities in well-being, which can inform targeted policy interventions. Prefectures without adjacent regions, such as Hokkaido and Okinawa, were excluded from this analysis.

To predict the RWI, we used the Elastic Net methodology, a machine-learning technique, that was designed to prevent the problems of multicollinearity and overfitting in a linear regression through regularization [34]. L1 regularization (lasso regression) applies a penalty to the absolute values of the coefficients, playing a role in excluding unnecessary variables from the model. L2 regularization (ridge regression) applies a penalty to the squared coefficients, thereby reducing the coefficients of highly correlated explanatory variables to overcome multicollinearity. The Elastic Net combines these 2 types of regularization, enabling the creation of a model that addresses the multicollinearity between explanatory variables and selects important features. To further prevent overfitting, we estimated model parameters using data from 2010, 2013, and 2016, and then assessed the prediction errors with 2019 data. This approach enabled us to evaluate the model’s performance on out-of-sample predictions, ensuring that it mitigates overfitting.

In this model, RSVs and prefectures served as predictor variables (with prefectures coded as dummy variables), whereas the IRWI score was used as the outcome variable. The performance of Elastic Net varies with 2 parameters: α, the mix ratio of L1 and L2 regularization, and λ, the strength of regularization. The optimal values for α and λ were determined by fixing α and identifying the λ that minimized the mean squared error through 10-fold cross-validation. This procedure was repeated 11 times, incrementally adjusting α from 0 to 1 by 0.1, and the model with the lowest mean squared error was selected. The predictive accuracy of the model was assessed using the root mean square error (RMSE) and the coefficient of determination (*R*^2^). All statistical analyses, including standard normalization, Pearson correlation coefficient calculation, and Elastic Net processing, were conducted using R software (version 4.1.3, The R Foundation).

### Ethical Considerations

This study used publicly accessible data from Google Trends and open government statistics for secondary use to eliminate the need for ethical review.

## Results

### Summary of RWI Scores

The median IRWIs by prefecture in Japan were 0.67 (IQR −2.48 to 2.71), 0.00 (IQR −2.85 to 2.76), 0.13 (IQR −3.05 to 2.49), and 0.19 (IQR −2.75 to 3.06) for 2010, 2013, 2016, and 2019, respectively (Table 1) [30]. The RWIs by 11 domains were also shown in Multimedia Appendices 2-5.

**Figure 2:**
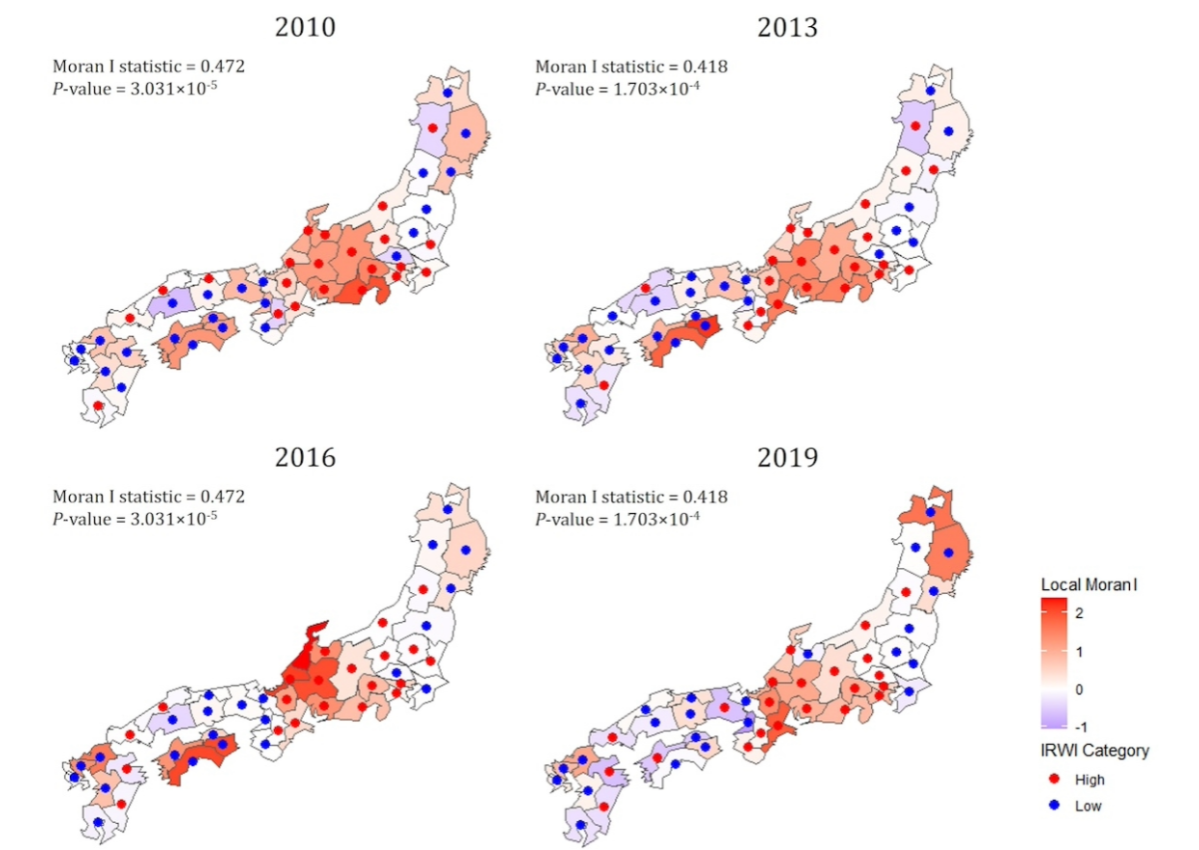
Spatial distribution of IRWI scores across Japan (2010-2019). IRWI scores are categorized as “High” or “Low” based on whether they are above or below the median. IRWI: integrated Regional Well-Being Index.

From 2010 to 2019, the Global Moran *I* statistics for the spatial analysis of IRWI scores across Japan ranged from 0.297 to 0.526, reflecting significant spatial autocorrelation throughout the entire period (Figure 2). Corresponding *P* values varied from 4.955 × 10⁻³ to 3.892 × 10⁻⁶, indicating strong statistical significance. For Local Moran *I*, median values in 2010, 2013, 2016, and 2019 were 0.333 (IQR 0.036-0.858), 0.214 (IQR 0.006-0.746), 0.113 (IQR –0.020 to 0.825), and 0.146 (IQR ‒0.055 to 0.829), respectively. Figure 2 shows the spatial distribution of Local Moran *I* scores, with regions color-coded according to their values for each year. Additionally, each prefecture is marked with red or blue dots to indicate whether their IRWI scores were above or below the median. This visualization showed the spatial clustering of regions with high or low IRWI scores.

**Table 1:** Integrated scores of the Regional Well-Being Index. All scores were standardized and unitless.

Prefecture	Year			
	2010	2013	2016	2019
Hokkaido	−2.02	−2.66	−4.28	−4.46
Aomori	−4.13	−6.69	−2.48	−6.45
Iwate	−4.46	−3.82	−3.86	−7.35
Miyagi	−4.55	1.85	−2.77	−2.49
Akita	2.21	3.08	−0.74	0.19
Yamagata	0.91	1.90	1.22	2.04
Fukushima	−1.62	−1.68	−3.27	−1.14
Ibaraki	2.51	−0.43	0.55	0.27
Tochigi	0.67	−0.66	1.05	−1.91
Gunma	2.93	2.32	0.58	0.79
Saitama	−1.26	0.00	−0.22	0.53
Chiba	1.54	1.31	0.42	−1.80
Tokyo	2.30	1.74	5.82	4.80
Kanagawa	1.30	2.79	0.82	3.14
Niigata	1.76	1.62	2.54	2.98
Toyama	3.95	0.95	5.20	−0.20
Ishikawa	4.04	2.42	6.30	5.50
Fukui	4.22	6.52	9.67	4.31
Yamanashi	8.14	7.83	6.91	5.77
Nagano	5.36	4.92	1.85	2.60
Gifu	4.52	6.45	7.45	5.52
Shizuoka	6.62	5.34	4.24	3.81
Aichi	3.80	4.74	5.99	4.12
Mie	2.61	7.41	5.95	7.30
Shiga	4.31	2.93	4.13	5.81
Kyoto	−2.88	−3.08	−0.36	−0.73
Osaka	−8.97	−10.18	−7.35	−7.97
Hyogo	−3.27	−2.95	−0.03	2.30
Nara	2.81	5.67	1.04	4.57
Wakayama	−0.30	2.72	0.13	2.76
Tottori	2.00	−1.25	−0.43	−4.44
Shimane	6.28	3.56	3.35	−0.54
Okayama	−2.38	−1.39	−4.01	−4.63
Hiroshima	−2.32	−4.22	−2.48	−2.36
Yamaguchi	1.33	0.12	2.43	3.14
Tokushima	−2.58	−4.79	−4.00	−1.76
Kagawa	−3.98	−1.38	−2.01	−3.01
Ehime	−3.30	−3.19	−4.06	2.69
Kochi	−5.27	−7.26	−8.56	−3.58
Fukuoka	−6.88	−7.70	−10.52	−5.74
Saga	0.03	−1.85	−4.11	−3.36
Nagasaki	−1.66	−2.12	−0.10	−0.83
Kumamoto	−1.34	−2.84	−4.43	−7.07
Oita	−2.17	−1.23	1.21	2.92
Miyazaki	−0.97	1.85	1.07	2.52
Kagoshima	1.06	−2.86	−2.82	−1.82
Okinawa	−10.91	−5.81	−7.05	−6.77
Overall, median (IQR)	0.67 (−2.48 to 2.71)	0.00 (−2.85 to 2.76)	0.13 (−3.05 to 2.49)	0.19 (−2.75 to 3.06)

### Characteristics of Words and Their RSVs

**Figure 3:**
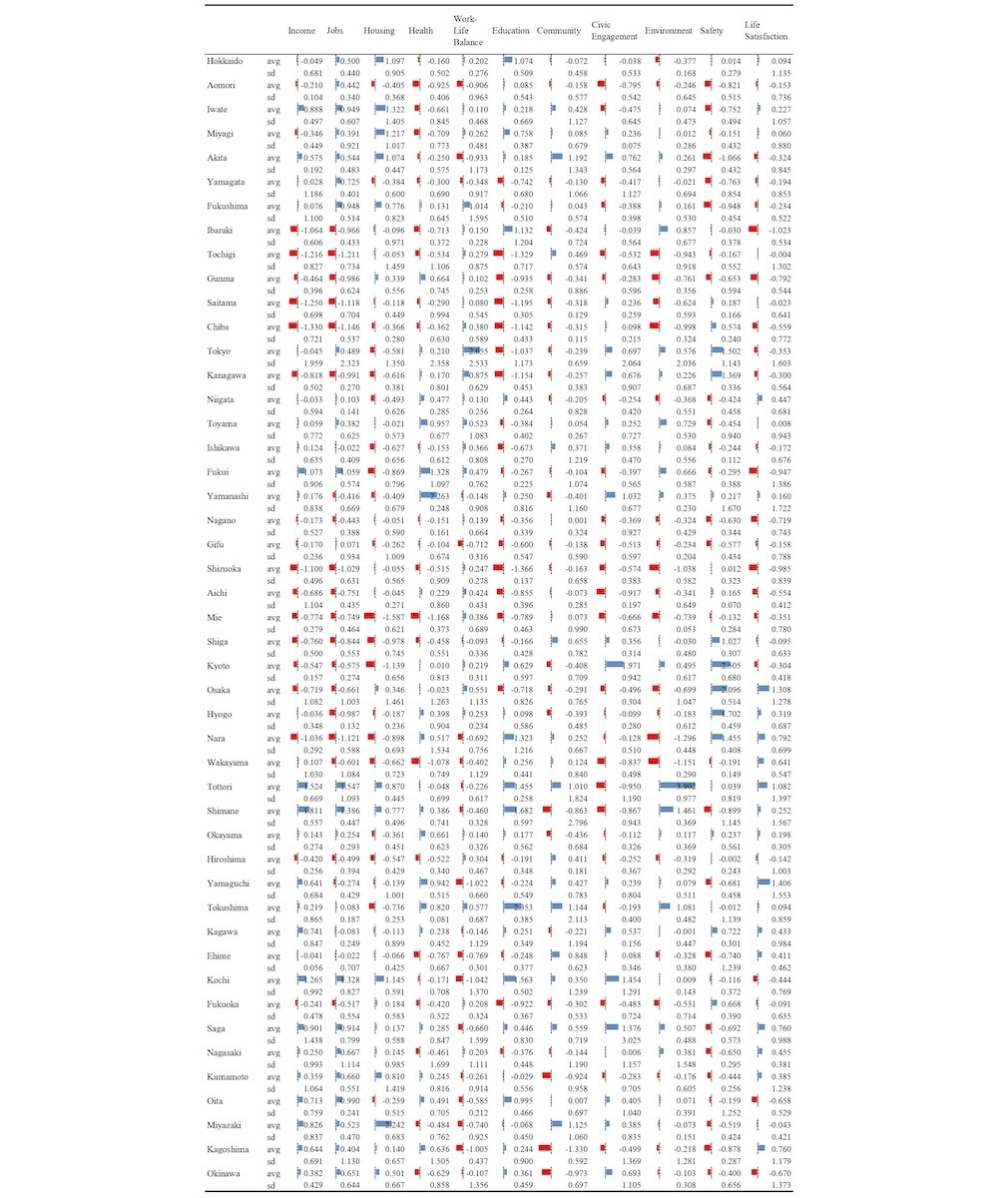
Temporal variations in regional relative search volumes: Descriptive statistics of mean and SD. “avg” is the average relative search volumes for 2010, 2013, 2016, and 2019. “sd” is the SD of relative search volumes in 2010, 2013, 2016, and 2019.

Eleven representative words were extracted for each domain, following a previous study [30], and 275 related words were associated with these representative words. Of these, the RSVs for 211 words were collected and those that met the exclusion criteria were excluded (Figure 1 and Multimedia Appendix 6). The mean search frequencies for the representative words of each domain during the data collection period (2010, 2013, 2016, and 2019) ranged from −1.587 to 3.902, with SDs ranging from 3.025 to 0.053 (Figure 3).

The partial correlation coefficients between each domain of the RWI and the RSV of each word varied in ranges, indicating the minimum and maximum extents of correlation. Specifically, the coefficients ranged as follows: for “Income,” from −0.301 to 0.226; for “Jobs,” from −0.315 to 0.133; for “Housing,” from −0.604 to 0.225; for “Health,” from −0.283 to 0.297; for “Work-Life Balance,” from −0.285 to 0.350; for “Education,” from −0.396 to 0.269; for “Community,” from −0.216 to 0.063; for “Civic Engagement,” from −0.233 to 0.269; for “Environment,” from −0.070 to 0.261; for “Safety,” from −0.183 to 0.036; and for “Life Satisfaction,” from −0.112 to 0.219, as detailed in Multimedia Appendix 6. Additionally, the overall range for the partial correlation coefficient between the IRWI and RSV for each word was determined to be from −0.409 to 0.362, as also noted in Multimedia Appendix 6.

### Prediction Model Using Search Log Data and Elastic Net

The best Elastic Net model was constructed using training data from 2010 to 2016, incorporating 2 to 13 variables per domain (α=0.1, λ=0.906, RMSE=1.290, and *R*^2^=0.940). This model was then used to predict outcomes for the 2019 test data, yielding an RMSE of 2.328 and an *R*^2^ of 0.665 (Tables 2 and 3). The model included 2-13 variables per domain as features. The standardized partial regression coefficients for words ranged from −0.386 to 0.489, and for the selected prefectures as features, they ranged from −0.704 to 0.439 (Tables 2 and 3).

**Table 2:** Elastic Net regression analysis metrics.

Index	Words selected for the model (standardized partial regression coefficient)^a^
Intercept	4.078×10^−15^
Income	給与 (−0.063), 所得 申告 (−0.006), 所得 証明 (−0.008), 所得 証明 書 (−0.021), 確定 申告 (0.489), 扶養 所得 (0.030), 譲渡 所得 (0.117), 退職 所得 (0.028), 住民 税 (0.013)
Jobs	雇用 契約 (0.226), 雇用 保険 証 (0.020), 雇用 保険 者 証 (−0.125), 雇用 保険 被 保険 者 証 (0.043), 助成 金 雇用 (−0.104), 雇用 保険 料率 (−0.011), 失業 保険 (−0.386)
Housing	住宅 (−0.116), 住宅 控除 (0.087), 賃貸 住宅 (−0.143), 市営 住宅 (−0.107), 住宅 ロ−ン 控除 (−0.046), 注文 住宅 (0.033), マンション (−0.264), 住宅 展示 場 (−0.127), 住宅 情報 (−0.381), 高齢 者 住宅 (−0.025), リフォ−ム (0.059), 分譲 住宅 (0.299), エコ ポイント 住宅 (0.171)
Health	健康 保険 組合 (0.360), 健康 センタ− (0.035), 健康 ランド (0.320), 健康 管理 (−0.048), 健康 保険 協会 (−0.026), 保険 証 (−0.069), 全国 健康 保険 協会 (−0.275), 健康 保険 とは (−0.135), 健康 管理 センタ− (0.075)
Work-life balance	残業 手当 (−0.018), 労働 時間 (−0.164), 労働 基準 法 (0.001), 転職 (0.130), 月 残業 時間 (−0.159)
Education	教育 (−0.118), 教育 大学 (−0.068), 教育 委員 会 (−0.338), 特別 教育 (−0.145), 教育 指導 (−0.247), 教育 ロ−ン (−0.179), 教育 研究 所 (0.055), 義務 教育 (0.351), 教育 問題 (0.113)
Community	疲れ た (−0.019), 人間関係 悩み (−0.053), 人間 関係 苦手 (0.141)
Civic engagement	政治 (−0.048), 政治 ブログ (−0.307), 選挙 (0.250), 政治 問題 (−0.160)
Environment	環境 基準(−0.330), 自然 環境 (0.259)
Safety	治安 (−0.134), 日本 治安 (−0.016)
Life satisfaction	幸せ (−0.063), 幸せ の 時間 (−0.209), 幸せ 画像 (−0.007), 幸せ に なりたい (−0.133), 幸せ に なろう (−0.154), 幸せ に なるために (−0.034), 小さな 幸せ (−0.094)
Prefecture	Aomori (−0.260), Iwate (−0.246), Akita (0.010), Fukushima (−0.081), Saitama (−0.031), Tokyo (0.167), Toyama (0.033), Ishikawa (0.126), Fukui (0.275), Yamanashi (0.319), Nagano (0.089), Gifu (0.439), Shizuoka (0.323), Aichi (0.415), Mie (0.334), Shiga (0.243), Osaka (−0.704), Shimane (0.269), Okayama (−0.171), Hiroshima (−0.159), Yamaguchi (0.050), Tokushima (−0.188), Kagawa (−0.245), Kochi (−0.232), Fukuoka (−0.505), Kumamoto (−0.069), Miyazaki (0.044), Okinawa (−0.497)

^a^Please refer to Multimedia Appendix 6 for English translations of Japanese words.

**Table 3:** Model accuracy statistics.

	Training data (2010-2016)	Test data (2019)
α^a^	0.1	—^b^
λ^c^	0.906	—
RMSE^d^	1.290	2.328
*R^2e^*	0.904	0.665

^a^α represents the proportion of L1 to L2 regularization in Elastic Net. This value is calculated using the training data and is part of the model used to predict the test data but is not recalculated for the test data.

^b^Not applicable.

^c^λ represents the intensity of regularization. This value is calculated using the training data and is part of the model used to predict the test data but is not recalculated for the test data.

^d^RMSE: root mean square error; the square root of the mean squared error.

^e^*R*^2^ (coefficient of determination) is the determination coefficient’s value.

## Discussion

### Principal Findings

The primary aim of this study was to predict the RWI for each prefecture in Japan using internet search log data. The best model in this study achieved an out-of-sample *R*^2^ value of 0.665 (in-sample *R*^2^ of 0.904; Tables 2 and 3), which is relatively high compared with the *R*^2^ values ranging from 0.005 to 0.830 reported in previous regional-level well-being studies using web data (Table 4) [27,28,35-39]. Most previous studies have focused on predicting subjective well-being or single-objective indicators, representing only one aspect of well-being. Unlike earlier research, this study’s comprehensive approach to predicting the RWI underscores the efficacy of internet search data in evaluating overall well-being.

**Table 4:** Previous studies predicting well-being-related indicators using web data.

Levels and reference			Outcomes	Big Data measure		Big Data source		*R* ^2a^
**Individuals**								
	Kosinski et al [35]	Life satisfaction		Type of Facebook pages liked	Facebook		0.003	
	Schwartz et al [36]	Life satisfaction		Posts	Facebook		0.090	
	Panicheva et al [37]	Life satisfaction		Message, Posts, App Usage, App Time, Number of friends	VKontakte^b^		0.116	
	Panicheva et al [37]	Psychological well-being		Message, App Usage, App Time, Number of friends, Posts	VKontakte		0.119	
**National**								
	Algan et al [28]	Life evaluation today		Word searches	Google Trends		0.940	
	Algan et al [28]	Life evaluation in 5 years		Word searches	Google Trends		0.840	
	Algan et al [28]	Happiness		Word searches	Google Trends		0.590	
	Carpi et al [38]	Human Development Index		Tweets	Twitter		0.640	
**State**								
	Durahim and Coskun [39]	Happiness		Tweets	Twitter		0.005	
	Algan et al [28]	Life evaluation today		Word searches	Google Trends		0.720	
	Algan et al [28]	Life evaluation in 5 years		Word searches	Google Trends		0.820	
	Algan et al [28]	Happiness		Word searches	Google Trends		0.830	
	Jaidka et al [27]	Life satisfaction		Tweets	Twitter		0.384	
	Jaidka et al [27]	Happiness		Tweets	Twitter		0.260	

^a^*R*^2^ (coefficient of determination) is the determination coefficient’s value.

^b^“VKontakte” is Russia’s social networking platform.

The primary advantage of this study is its potential to reduce the time and economic resources required for conventional well-being assessments significantly. The developed model allows for frequent and quick assessment of comprehensive well-being at the prefectural level in Japan using upcoming search log data. This approach could be invaluable for policymaking aimed at enhancing well-being. Moreover, this methodology can be adapted beyond Japan, allowing countries where the BLI is measured to calculate their RWI based on the Japanese approach and to predict regional well-being using web data based on this study's methods. If the accuracy of the model is ensured, it will allow immediate and repeated assessments of comprehensive well-being. Additionally, the use of Google, a platform extensively used globally, in this study suggests its potential applicability even in many resource-limited nations where large-scale surveys pose challenges. The methodology proposed in this study could be tested in various countries, potentially enabling the assessment of well-being and the realization of evidence-based policy making (EBPM) for well-being improvement.

The analysis revealed marked disparities in mean search frequencies and their SDs when segmented by prefecture (Tables 2 and 3). These differences indicate variations in interest in well-being-related topics across regions. Notably, the tendency for higher search frequencies related to work-life balance in major urban areas, such as Tokyo and Osaka, might suggest an increased awareness of the working environment in these regions. Furthermore, the magnitude of the SDs reflects the degree of variability in search behavior during the observation period, suggesting the potential to assess changes in the interests and states of well-being among residents of different regions.

In the analysis of the relationship between each word and regional well-being indicators (RWI) as well as IRWI in this study, correlation coefficients showed both positive and negative values, with many remaining within the range of ±0.3. Notably, in the “Housing” domain, there were relatively high positive correlations, such as a correlation coefficient of 0.6 for “マンション.” A positive correlation coefficient suggests that as the search frequency for a word increases, so does the RWI domain or IRWI score. Although these correlations do not establish causality, they indicate a potential relationship between search behaviors related to specific words and the well-being of people in that area or with more comprehensive well-being. This implies that internet search data could be a viable means of understanding well-being.

As a supplementary analysis, Global Moran *I* results indicate spatial autocorrelation in Japan’s IRWI scores, suggesting that well-being in Japan is not randomly distributed but exhibits spatial patterns. Additionally, Local Moran *I* indicated these spatial patterns specifically form. High-score clusters were predominantly found in the Chubu and Tokai regions, while low-score clusters were identified in parts of the Tohoku and Kyushu regions, as well as in Shikoku. This suggests that regional differences in well-being may not be solely due to isolated factors but could also be influenced by interrelationships with adjacent areas, possibly reflecting common factors across multiple regions. This finding could provide important implications for policy interventions aimed at improving well-being.

### Limitations

While focusing on predicting a comprehensive well-being index (IRWI), this study did not consider the causal relationship between fluctuations in search log data and changes in each well-being indicator of the RWI. Although the out-of-sample *R*^2^ and RMSE indicate a high degree of accuracy, the model might exhibit slight overfitting. This may be due to the limited data available, as the evaluation is restricted to a single year (2019). The RWIs used as outcomes in this study are based on somewhat outdated data, with 2019 being the most recent year available due to the unavailability of certain indicators constituting the RWI. Subsequent updates have been delayed by factors such as the COVID-19 pandemic and extended data aggregation processes. However, the year-to-year variations in RWIs are typically not substantial, suggesting that insights derived from data up to 2019 remain valuable and pertinent. Furthermore, including data from 2020 onward would introduce significant confounding effects due to the pandemic’s impact. Thus, the examination of these postpandemic trends and their implications remains an important issue for future research. Additionally, since this was an ecological study that used data at the prefecture-level rather than at the individual level, we could not evaluate associations at the individual level. It also did not address the correlations with specific RWI domains or the relative importance of each domain. The well-being indicators, BLI and RWI, are designed to allow users to assess the significance of each domain in a flexible manner. Therefore, the results of this study, which treated each domain uniformly, may differ from the interpretation of the RWI when used in a prefecture. Consequently, implementation in the field should be a topic for future research. Another limitation is the lack of consideration for the emotional valence of search queries and the reason why certain specific words significantly correlated with well-being. Although RSV captures public interest—which implicitly includes emotional aspects—the technical challenges of applying natural language processing techniques to individual search terms limited their use in this study. Therefore, future research is warranted to better understand regional characteristics and to derive more accurate interpretations of searchers’ intentions from search terms associated with well-being scores. Addressing these challenges could provide deeper insights into the relationship between web-based behavior and well-being.

In addition to these limitations, Google Trends data was limited by its relative nature, which might not have fully captured actual search activity. While it is possible that infrequently searched words would be overestimated, we believe that the impact is limited in this study because niche words were excluded during the process of extracting representative words. Additionally, the algorithms behind Google’s search functions introduced uncertainty in interpreting search intent [40]. It was also noted that the granularity of Google Trends data could have led to limitations in predictive accuracy [41].

### Comparison With Prior Work

This study successfully improved the predictive accuracy of comprehensive well-being indices at the regional level through the use of web data, achieving relatively higher accuracy compared with the outcomes of previous studies, as shown in Table 4. Kosinski et al [35] and Schwartz et al [36], used Facebook data and predicted individual-level life satisfaction but achieved relatively low *R*^2^ values of 0.003 and 0.090, respectively. Panicheva et al [37] integrated various types of web data, including messages and attributes from VKontakte, a SNS popular in Russia and its neighboring countries, to predict individual-level life satisfaction and mental well-being; however, the improvement in *R*^2^ was limited.

In contrast, predictions at broader national and state levels yielded more accurate results. A notable example is Algan et al [28], who achieved high predictive accuracy with *R*^2^ values of 0.940 at the national level and 0.720 at the state level for Life Satisfaction. Our study showed similar levels of accuracy in forecasting a more comprehensive well-being index, effectively capturing trends in regional well-being.

Furthermore, the predictive model in this study demonstrated a level of accuracy comparable to that reported by Carpi et al [38], who predicted the Human Development Index, a comprehensive composite index analogous to the RWI used in this research. This result suggests that our methodology can efficiently and accurately predict comprehensive well-being indicators using web data, suggesting that it can quickly identify broader well-being trends while saving time and money.

### Implications and Actions Needed

Using the RWI calculation methodology of this study, OECD countries have the opportunity to calculate the RWI for their regions and apply these in regional policymaking. Moreover, the creation of high-accuracy models using internet search data facilitates the timely and continuous assessment of well-being. Governments and international organizations are shifting their focus from merely economic development and life expectancy to improving well-being; however, integrating well-being indicators into national and regional policy goals is traditionally time-consuming and costly. This study demonstrates the efficacy of our approach in predicting comprehensive well-being indicators through web data, indicating its potential to rapidly assess broader trends in well-being with cost and time efficiency. Furthermore, these internet search data are likely to be less susceptible to biases common in traditional survey methods, such as recall and social desirability biases, possibly unveiling aspects of well-being that conventional approaches overlook. Comparing this approach with traditional methodologies may yield insights into societal well-being and provide foundational data for policymaking and evaluations aimed at improving well-being. If this approach is effective, it could extend the reach of well-being assessments to additional countries and regions, thereby accelerating the adoption of policies designed to improve societal well-being.

### Conclusion

This study predicted RWIs for Japanese prefectures with high accuracy using RSVs from internet search engines and an Elastic Net machine learning method. This approach provides an immediate and cost-effective alternative to traditional survey methods for comprehensive well-being assessments. It enables ongoing and quick assessments and serves as foundational data for EBPM focused on enhancing well-being. Adding the well-being indicators predicted by the method proposed in this study to conventional policy indicators will enable the agile detection of changes in the population and provide basic data for the discovery of new health issues and policy formulation. Moreover, this methodology suggests a potential solution for assessing well-being in resource-limited nations and regions, where large-scale epidemiological surveys are impractical. However, this study reflects ecological trends rather than individual behaviors, and further research is warranted to identify causal relationships between individual search terms and well-being.

## Appendix

Multimedia Appendix 1 Comparison of search frequencies for "人間関係" (human relationship) and "人間 関係" on Google Trends.

Multimedia Appendix 2 Scores of the Regional Well-Being Index for the year 2010.

Multimedia Appendix 3 Scores of the Regional Well-Being Index for the year 2013.

Multimedia Appendix 4 Scores of the Regional Well-Being Index for the year 2016.

Multimedia Appendix 5 Scores of the Regional Well-Being Index for the year 2019.

Multimedia Appendix 6 Representative words in the Better Life Index domains and related words for each domain.

## Abbreviations

BLI: Better Life Index
EBPM: evidence-based policy making
GDP: gross domestic product
IRWI: integrated Regional Well-Being Index
OECD: Organization for Economic Co-operation and Development
RWI: Regional Well-Being Index
RSV: relative search volume
RMSE: root mean square error
SNS: social networking service

## References

[1] (2024). Health-related quality of life (HRQOL): well-being concepts. Centers for Disease Control and Prevention.

[2] Lee MT, Kubzansky LD, VanderWeele TJ (2021). Measuring Well-Being: Interdisciplinary Perspectives from the Social Sciences and the Humanities.

[3] Diener E, Seligman MEP (2004). Beyond money: toward an economy of well-being. Psychol Sci Public Interest.

[4] Diener E, Seligman MEP (2006). Measure for measure: the case for a national well-being index. Sci Spirit.

[5] Kahneman D, Krueger AB, Schkade D, Schwarz N, Stone A (2004). Toward national well-being accounts. Am Econ Rev.

[6] Linton MJ, Dieppe P, Medina-Lara A (2016). Review of 99 self-report measures for assessing well-being in adults: exploring dimensions of well-being and developments over time. BMJ Open.

[7] Steptoe A, Deaton A, Stone AA (2015). Subjective wellbeing, health, and ageing. Lancet.

[8] Stiglitz J, Sen A, Fitoussi JP (2009). Report by the commission on the measurement of economic performance and social progress. INSEE.

[9] Kakwani N (1993). Performance in living standards. J Dev Econ.

[10] Twenge JM (2000). The age of anxiety? birth cohort change in anxiety and neuroticism, 1952-1993. J Pers Soc Psychol.

[11] Diener E, Tov W, Land KC, Michalos AC, Sirgy MJ (2012). Handbook of Social Indicators and Quality of Life Research.

[12] (2011). How's life?: measuring well-being. Organization for Economic Co-operation and Development.

[13] Helliwell JF, Layard R, Sachs JD, Aknin LB, de Neve NE, Wang S World Happiness Report.

[14] (2023). Human development index. United Nations Development Programme.

[15] New Zealand Treasury (2023). Living Standards Framework Dashboard.

[16] (2023). UK measures of national well-being dashboard. Office for National Statistics.

[17] (2024). National wealth indicators. French National Institute for Statistics Economic Studies.

[18] (2024). Measuring-well-being. National Institute of Statistics.

[19] Diener E, Emmons RA, Larsen RJ, Griffin S (1985). The satisfaction with life scale. J Pers Assess.

[20] Lyubomirsky S, Lepper HS (1999). A measure of subjective happiness: preliminary reliability and construct validation. Soc Indic Res.

[21] OECD regional well-being. Organization for Economic Co-operation and Development.

[22] Eichstaedt JC, Schwartz HA, Kern ML, Park G, Labarthe DR, Merchant RM, Jha S, Agrawal M, Dziurzynski LA, Sap M, Weeg C, Larson EE, Ungar LH, Seligman MEP (2015). Psychological language on Twitter predicts county-level heart disease mortality. Psychol Sci.

[23] Llorente A, Garcia-Herranz M, Cebrian M, Moro E (2015). Social media fingerprints of unemployment. PLoS One.

[24] Mendoza M, Poblete B, Valderrama I (2019). Nowcasting earthquake damages with Twitter. EPJ Data Sci.

[25] Kadar C, Rosés BR, Pletikosa I (2017). Measuring ambient population from location-based social networks to describe urban crime.

[26] Xie Z, Liu G, Wu J, Wang L, Liu C (2016). Wisdom of fusion: prediction of 2016 Taiwan election with heterogeneous big data.

[27] Jaidka K, Giorgi S, Schwartz HA, Kern ML, Ungar LH, Eichstaedt JC (2020). Estimating geographic subjective well-being from Twitter: a comparison of dictionary and data-driven language methods. Proc Natl Acad Sci U S A.

[28] Algan Y, Murtin F, Beasley E, Higa K, Senik C (2019). Well-being through the lens of the internet. PLoS One.

[29] Google Trends.

[30] Yang MS, Taira K (2024). Calculating a prefecture-level well-being index in Japan: applying the framework of the OECD's better life index. Nihon Koshu Eisei Zasshi.

[31] Ministry of Internal Affairs and Communications (2020). Population Census.

[32] Moran PAP (1950). Notes on continuous stochastic phenomena. Biometrika.

[33] Anselin L (2010). Local indicators of spatial association—LISA. Geogr Anal.

[34] Hui Z, Trevor H (2005). Regularization and variable selection via the elastic net. J R Stat Soc Series B Stat Methodol.

[35] Kosinski M, Stillwell D, Graepel T (2013). Private traits and attributes are predictable from digital records of human behavior. Proc Natl Acad Sci U S A.

[36] Schwartz HA, Sap M, Kern ML, Eichstaedt JC, Kapelner A, Agrawal M, Blanco E, Dziurzynski L, Park G, Stillwell D, Kosinski M, Seligman ME, Ungar L (2016). Predicting individual welk-being through the language of social media. Pac Symp Biocomput.

[37] Panicheva P, Mararitsa L, Sorokin S, Koltsova O, Rosso P (2022). Predicting subjective well-being in a high-risk sample of russian mental health app users. EPJ Data Sci.

[38] Carpi T, Hino A, Iacus S, Porro G (2022). A Japanese subjective well-being indicator based on Twitter data. Soc Sci Jpn J.

[39] Durahim AO, Coşkun M (2015). #iamhappybecause: gross national happiness through Twitter analysis and big data. Technol Forecast Soc Change.

[40] Bilić P (2016). Search algorithms, hidden labour and information control. Big Data Soc.

[41] Jun SP, Yoo HS, Choi S (2018). Ten years of research change using google trends: from the perspective of big data utilizations and applications. Technol Forecast Soc Change.

[42] DeepL.

[43] ChatGPT. OpenAI.

