# Supplementary material for: Predicting Prefecture-Level Well-Being Indicators in Japan Using Search Volumes in Internet Search Engines: Infodemiology Study
Source: J Med Internet Res. 2024 Nov 11;26:e64555. doi: 10.2196/64555 (PMC11589491; doi:10.2196/64555)
Supplement: Multimedia Appendix 1 [file jmir_v26i1e64555_app1.docx]

**Multimedia Appendix 1. Comparison of Search Frequencies for "人間関係 (Human Relationship)" and "人間 関係" on Google Trends**

Note: "*r*" represents the Pearson correlation coefficient.
