# Supplementary material for: Predicting Prefecture-Level Well-Being Indicators in Japan Using Search Volumes in Internet Search Engines: Infodemiology Study
Source: J Med Internet Res. 2024 Nov 11;26:e64555. doi: 10.2196/64555 (PMC11589491; doi:10.2196/64555)
Supplement: Multimedia Appendix 2 [file jmir_v26i1e64555_app2.docx]

**Multimedia Appendix 2. Scores of the Regional Well−Being Index for the Year 2010**

| Prefecture | **Income** | **Jobs** | **Housing** | **Health** | **Work−Life Balance** | **Education** | **Community** | **Civic Engagement** | **Environment** | **Safety** | **Life Satisfaction** |
| --- | --- | --- | --- | --- | --- | --- | --- | --- | --- | --- | --- |
| Hokkaido | −0.58 | −0.40 | −1.05 | −0.57 | −0.14 | −0.62 | 0.37 | 0.63 | 0.00 | 0.09 | 0.24 |
| Aomori | −0.95 | −2.05 | 0.61 | −2.14 | −1.14 | 0.61 | −0.64 | −1.19 | 1.67 | 2.05 | −0.96 |
| Iwate | −1.09 | −0.40 | 0.73 | −1.44 | −1.32 | −0.67 | −2.00 | 0.25 | 1.67 | 1.27 | −1.46 |
| Miyagi | −0.65 | −1.11 | −0.67 | −0.03 | 0.31 | 0.16 | −0.39 | −1.49 | 0.00 | −0.62 | −0.09 |
| Akita | −1.05 | −0.75 | 1.30 | 0.06 | −1.08 | 2.13 | −1.03 | 1.41 | 1.67 | 0.99 | −1.43 |
| Yamagata | −0.83 | 0.31 | 1.27 | 0.53 | −0.67 | 0.42 | −0.96 | 1.15 | 0.00 | 0.81 | −1.12 |
| Fukushima | −0.40 | −0.52 | 0.34 | −0.65 | −0.73 | −0.45 | 0.04 | 0.56 | 0.00 | 0.38 | −0.19 |
| Ibaraki | 0.75 | −0.04 | −0.03 | 1.32 | 0.93 | −0.13 | −1.45 | −1.05 | 1.67 | −0.07 | 0.63 |
| Tochigi | 0.95 | 0.08 | −0.07 | 0.46 | −0.61 | −0.26 | −1.21 | −0.69 | 0.00 | 0.86 | 1.16 |
| Gunma | 0.40 | 0.08 | −0.11 | 0.95 | −0.18 | 0.81 | −0.08 | −0.20 | 0.00 | 0.19 | 1.07 |
| Saitama | 0.33 | −0.52 | −1.10 | 0.37 | 1.70 | −0.30 | −0.42 | −0.87 | −0.58 | −0.46 | 0.58 |
| Chiba | 0.46 | 0.08 | −1.04 | 1.76 | 1.99 | −0.09 | −0.24 | −1.12 | −0.58 | −0.83 | 1.15 |
| Tokyo | 4.55 | −0.87 | −2.68 | −0.62 | 0.42 | 0.32 | 0.84 | −0.16 | 0.00 | −0.03 | 0.53 |
| Kanagawa | 0.59 | −0.16 | −1.75 | 0.70 | 1.89 | −0.14 | 0.27 | −0.94 | −0.58 | 0.53 | 0.89 |
| Niigata | −0.21 | 0.43 | 1.08 | −0.74 | −0.40 | −0.20 | −0.10 | 0.41 | 1.67 | 0.55 | −0.72 |
| Toyama | 0.94 | 1.14 | 2.05 | 0.31 | −0.54 | 1.51 | −0.46 | 1.37 | −0.58 | 0.38 | −2.16 |
| Ishikawa | 0.24 | 0.55 | 0.79 | 1.00 | −0.34 | 1.05 | 0.16 | 0.12 | −0.58 | 1.10 | −0.05 |
| Fukui | 0.48 | 1.73 | 1.63 | 1.01 | −1.06 | 2.39 | −2.08 | 1.47 | −0.58 | −0.39 | −0.38 |
| Yamanashi | 0.25 | 0.43 | 0.07 | 1.14 | 0.17 | −0.20 | 1.97 | 1.16 | 1.67 | 0.19 | 1.27 |
| Nagano | −0.13 | 0.90 | 0.78 | 1.10 | −0.09 | −0.71 | 0.19 | 1.33 | 1.67 | −0.44 | 0.76 |
| Gifu | −0.10 | 1.26 | 1.31 | 0.69 | −0.01 | 1.09 | −0.07 | 0.10 | −0.58 | 0.76 | 0.08 |
| Shizuoka | 1.12 | 1.02 | −0.25 | 1.84 | −0.20 | 0.76 | 0.76 | −0.49 | 1.67 | −0.42 | 0.80 |
| Aichi | 1.10 | 0.55 | −0.64 | 1.93 | 0.70 | 0.33 | −0.52 | −0.47 | 0.00 | −0.06 | 0.85 |
| Mie | 0.69 | 0.90 | 0.75 | 0.46 | 0.97 | −0.23 | −1.20 | 0.37 | −0.58 | −0.01 | 0.50 |
| Shiga | 1.40 | 0.55 | 1.11 | 0.37 | 0.95 | 0.05 | −0.13 | 0.36 | 0.00 | 0.39 | −0.73 |
| Kyoto | 0.54 | −1.11 | −0.91 | −0.03 | 1.62 | −0.42 | 0.46 | −1.40 | −0.58 | −0.92 | −0.13 |
| Osaka | 0.58 | −2.53 | −1.61 | −1.50 | 0.74 | −1.39 | 0.60 | −0.75 | −0.58 | −2.53 | −0.02 |
| Hyogo | 0.12 | −0.63 | −0.51 | −0.68 | 1.79 | 0.25 | −0.45 | −1.23 | −0.58 | −1.27 | −0.08 |
| Nara | −0.52 | −0.04 | 0.72 | −0.05 | 2.40 | 0.38 | −0.57 | −0.06 | −0.58 | 0.13 | 1.00 |
| Wakayama | −0.15 | 0.55 | 0.28 | −0.01 | 0.97 | −0.71 | 0.15 | 0.01 | −0.58 | −1.17 | 0.35 |
| Tottori | −1.11 | 0.67 | 1.08 | −0.55 | −0.30 | 0.53 | −0.89 | 1.59 | 0.00 | 1.39 | −0.42 |
| Shimane | −1.02 | 1.85 | 1.18 | 0.05 | −0.07 | 0.38 | 0.01 | 3.06 | 0.00 | 1.60 | −0.75 |
| Okayama | −0.19 | 0.55 | 0.30 | −1.11 | −1.01 | −0.38 | 1.87 | −0.59 | −0.58 | −1.28 | 0.05 |
| Hiroshima | 0.53 | 0.79 | −0.46 | −0.29 | −0.24 | 0.08 | 0.42 | −1.45 | −0.58 | 0.18 | −1.30 |
| Yamaguchi | 0.43 | 1.02 | −0.02 | 0.08 | 0.27 | 0.35 | −0.91 | 0.63 | −0.58 | 0.68 | −0.62 |
| Tokushima | 0.18 | 0.08 | 0.35 | −0.76 | 0.07 | 0.33 | −1.18 | −0.28 | −0.58 | 0.82 | −1.61 |
| Kagawa | 0.08 | 1.02 | 0.65 | −0.81 | −0.44 | 0.75 | −1.36 | −0.41 | −0.58 | −1.53 | −1.36 |
| Ehime | −0.51 | 0.19 | −0.08 | −1.15 | −0.71 | 0.10 | 2.18 | −0.45 | −0.58 | −2.18 | −0.11 |
| Kochi | −0.98 | −0.28 | −0.28 | −1.89 | 0.33 | −1.81 | 1.46 | −0.21 | −0.58 | −0.11 | −0.91 |
| Fukuoka | 0.14 | −1.46 | −1.27 | −1.09 | −0.32 | −0.66 | 0.19 | −0.82 | 0.00 | −1.05 | −0.52 |
| Saga | −0.54 | 0.31 | 0.58 | −0.11 | −1.22 | −0.51 | 0.94 | 0.92 | −0.58 | 0.55 | −0.31 |
| Nagasaki | −0.88 | −0.28 | −0.34 | −1.87 | −2.36 | 0.47 | 1.09 | 0.48 | 0.00 | 1.59 | 0.44 |
| Kumamoto | −0.90 | −0.28 | −0.48 | 0.24 | −0.85 | 0.23 | 0.96 | 0.63 | −0.58 | −0.06 | −0.24 |
| Oita | −0.40 | 0.19 | −0.28 | −0.83 | −0.79 | −0.66 | 0.99 | 0.89 | −0.58 | 0.28 | −0.99 |
| Miyazaki | −1.19 | −0.16 | −0.67 | 0.94 | −0.83 | −0.02 | −0.13 | −0.64 | 0.00 | −0.43 | 2.17 |
| Kagoshima | −0.75 | −0.40 | −0.99 | 1.05 | 0.03 | −0.75 | 1.13 | −0.25 | 0.00 | 0.17 | 1.81 |
| Okinawa | −1.72 | −3.24 | −1.66 | 0.57 | −0.63 | −4.22 | 1.44 | −1.72 | 0.00 | −2.07 | 2.32 |
| Median (IQR) | −0.10 (−0.70 - 0.51) | 0.08 (−0.40 - 0.55) | −0.03 (−0.66 - 0.74) | 0.05 (−0.71 - 0.70) | −0.18 (−0.69 - 0.56) | 0.05 (−0.44 - 0.40) | −0.07 (−0.61 - 0.68) | −0.16 (−0.72 - 0.63) | 0.00 (−0.58 - 0.00) | 0.13 (−0.45 - 0.62) | −0.08 (−0.73 - 0.70) |
