# Supplementary material for: Predicting Prefecture-Level Well-Being Indicators in Japan Using Search Volumes in Internet Search Engines: Infodemiology Study
Source: J Med Internet Res. 2024 Nov 11;26:e64555. doi: 10.2196/64555 (PMC11589491; doi:10.2196/64555)
Supplement: Multimedia Appendix 4 [file jmir_v26i1e64555_app4.docx]

**Multimedia Appendix 4. Scores of the Regional Well−Being Index for the Year 2016**

| **Prefecture** | **Income** | **Jobs** | **Housing** | **Health** | **Work−Life Balance** | **Education** | **Community** | **Civic Engagement** | **Environment** | **Safety** | **Life Satisfaction** |
| --- | --- | --- | --- | --- | --- | --- | --- | --- | --- | --- | --- |
| Hokkaido | −0.48 | −1.60 | −1.05 | −0.11 | −0.12 | −0.41 | −1.71 | 0.47 | 0.58 | 0.18 | −0.03 |
| Aomori | −0.77 | −1.60 | 0.64 | −0.79 | −1.02 | 0.44 | −0.53 | 0.09 | 0.58 | 1.21 | −0.72 |
| Iwate | −0.55 | 0.46 | 0.73 | −0.39 | −1.43 | −1.10 | −1.24 | 0.72 | 0.58 | 0.22 | −1.86 |
| Miyagi | 0.10 | −0.85 | −0.72 | 0.62 | −0.31 | 0.01 | −0.12 | −0.66 | 0.58 | −0.51 | −0.91 |
| Akita | −0.79 | −0.48 | 1.26 | −1.63 | −0.73 | 2.25 | −1.66 | 1.52 | 0.58 | 0.85 | −1.92 |
| Yamagata | −0.43 | 0.65 | 1.38 | 1.09 | −1.16 | 0.01 | −1.14 | 1.87 | 0.58 | −0.10 | −1.54 |
| Fukushima | −0.03 | 0.08 | 0.35 | −0.99 | −1.45 | −0.97 | −0.70 | 0.55 | 0.58 | 0.54 | −1.25 |
| Ibaraki | 0.34 | −0.10 | 0.04 | 0.88 | 0.12 | 0.24 | −1.28 | −1.08 | 0.58 | −0.20 | 1.01 |
| Tochigi | 0.78 | 0.08 | −0.02 | 0.14 | −0.37 | −0.26 | 0.71 | −0.92 | 0.58 | −0.64 | 0.96 |
| Gunma | 0.61 | 0.46 | −0.09 | 0.04 | −0.10 | 0.72 | −0.97 | −1.15 | 0.58 | −0.02 | 0.49 |
| Saitama | 0.13 | −0.67 | −1.07 | 2.04 | 2.18 | −0.67 | −0.52 | −0.78 | −0.19 | −1.22 | 0.54 |
| Chiba | 0.21 | −0.29 | −1.04 | 0.62 | 1.57 | −0.40 | 0.42 | −0.76 | 0.44 | −0.80 | 0.46 |
| Tokyo | 5.52 | −0.85 | −2.72 | −0.09 | 0.88 | 0.71 | 0.51 | 0.65 | 0.46 | 0.05 | 0.72 |
| Kanagawa | 0.36 | −0.67 | −1.74 | 0.49 | 1.63 | −0.11 | −0.74 | 0.13 | 0.58 | −0.07 | 0.96 |
| Niigata | −0.09 | −0.10 | 1.09 | 0.78 | −0.67 | 0.14 | −1.22 | 1.24 | 0.58 | 0.68 | 0.13 |
| Toyama | 0.38 | 0.83 | 1.99 | 1.03 | −0.88 | 1.66 | −1.53 | 0.16 | 0.58 | 2.08 | −1.11 |
| Ishikawa | 0.02 | 1.21 | 0.81 | 1.21 | −0.61 | 1.79 | −0.20 | 0.49 | 0.58 | 0.69 | 0.32 |
| Fukui | 0.17 | 1.58 | 1.57 | 0.78 | −0.12 | 2.56 | 0.30 | 0.39 | 0.58 | 1.94 | −0.09 |
| Yamanashi | −0.12 | 0.83 | 0.15 | 2.26 | 0.37 | −0.27 | 1.02 | 0.99 | 0.58 | −0.48 | 1.57 |
| Nagano | −0.12 | 0.46 | 0.74 | 0.12 | −0.51 | −0.02 | −0.80 | 2.03 | 0.58 | −0.07 | −0.56 |
| Gifu | −0.03 | 1.21 | 1.30 | 1.65 | 1.16 | 0.51 | 0.75 | 0.71 | 0.58 | −0.35 | −0.05 |
| Shizuoka | 0.84 | 0.46 | −0.18 | 1.13 | −0.02 | 1.09 | 0.33 | 0.20 | 0.58 | −1.07 | 0.87 |
| Aichi | 1.58 | 0.65 | −0.65 | 1.96 | 0.59 | 0.69 | −0.29 | 0.11 | 0.58 | −0.79 | 1.55 |
| Mie | 0.24 | 1.40 | 0.66 | −0.70 | 0.37 | −0.49 | 0.93 | 1.23 | 0.58 | 1.11 | 0.62 |
| Shiga | 0.57 | 0.46 | 1.07 | 0.49 | 0.92 | −0.89 | 0.74 | 0.40 | 0.58 | 0.16 | −0.36 |
| Kyoto | −0.02 | −0.67 | −0.92 | −0.39 | 1.89 | 0.33 | −0.22 | −0.98 | 0.58 | 0.28 | −0.24 |
| Osaka | 0.01 | −2.35 | −1.61 | −1.05 | 1.20 | −0.79 | 0.40 | −0.70 | 0.11 | −2.60 | 0.03 |
| Hyogo | 0.05 | −1.23 | −0.54 | 0.06 | 2.20 | 0.80 | −0.93 | −0.32 | 0.13 | −0.71 | 0.44 |
| Nara | −0.52 | −0.48 | 0.72 | −1.26 | 2.65 | 0.21 | −0.94 | 0.50 | 0.58 | 0.36 | −0.77 |
| Wakayama | 0.10 | 1.02 | 0.35 | −1.34 | 0.33 | −0.80 | 1.14 | 0.08 | 0.16 | −0.52 | −0.38 |
| Tottori | −1.13 | 0.83 | 1.08 | −0.70 | −0.77 | 0.47 | −1.01 | 0.34 | 0.58 | 0.32 | −0.43 |
| Shimane | −0.23 | 1.96 | 1.13 | −0.66 | −0.65 | −0.49 | 0.58 | 1.86 | −0.17 | 0.70 | −0.68 |
| Okayama | −0.32 | 0.08 | 0.28 | −0.99 | −0.71 | −1.02 | 1.46 | −1.06 | −1.18 | −1.36 | 0.81 |
| Hiroshima | 0.54 | 0.08 | −0.49 | −0.15 | −0.29 | 0.32 | 0.30 | −1.39 | −0.75 | −0.12 | −0.55 |
| Yamaguchi | 0.49 | 0.65 | 0.01 | 0.26 | 0.12 | 0.70 | 0.80 | −0.42 | −1.22 | 1.00 | 0.04 |
| Tokushima | 0.21 | 0.08 | 0.42 | −1.38 | −0.75 | 0.36 | −0.87 | −2.06 | 0.58 | 0.83 | −1.42 |
| Kagawa | −0.07 | 0.27 | 0.57 | 0.62 | −0.24 | 0.77 | 0.33 | −1.27 | −1.92 | −0.47 | −0.61 |
| Ehime | −0.59 | 0.27 | −0.07 | −1.40 | −0.73 | 0.80 | 0.57 | 0.36 | −2.79 | 0.39 | −0.86 |
| Kochi | −0.67 | −1.04 | −0.28 | −1.32 | −0.14 | −1.12 | −0.47 | −2.43 | 0.58 | −0.93 | −0.74 |
| Fukuoka | −0.26 | −1.42 | −1.33 | −1.09 | −0.35 | −0.72 | 1.26 | −0.54 | −3.41 | −2.00 | −0.66 |
| Saga | −0.62 | 0.83 | 0.53 | −0.87 | −1.26 | −1.12 | −0.56 | 0.44 | −1.92 | 0.50 | −0.08 |
| Nagasaki | −0.69 | −0.10 | −0.34 | −0.42 | −0.94 | −0.32 | 2.33 | 0.24 | −0.47 | 0.97 | −0.36 |
| Kumamoto | −0.79 | −0.67 | −0.46 | 0.00 | 0.12 | 0.00 | 0.00 | −0.90 | −1.67 | −0.06 | 0.00 |
| Oita | −0.73 | 0.46 | −0.21 | −0.99 | −0.41 | −0.61 | 1.03 | 0.88 | −0.17 | 1.41 | 0.54 |
| Miyazaki | −1.06 | 0.83 | −0.66 | 0.02 | −0.69 | −0.59 | 1.45 | −1.34 | −0.08 | 0.98 | 2.21 |
| Kagoshima | −0.96 | −0.10 | −0.97 | 0.53 | −0.47 | −1.19 | 0.27 | 0.23 | −1.67 | 0.22 | 1.29 |
| Okinawa | −1.16 | −2.91 | −1.74 | −0.13 | −0.37 | −3.21 | 2.02 | −0.13 | 0.58 | −2.62 | 2.63 |
| Median (IQR) | −0.03 (−0.57 - 0.22) | 0.08 (−0.67 - 0.65) | 0.01 (−0.65 - 0.73) | 0.00 (−0.83 - 0.62) | −0.29 (−0.70 - 0.37) | 0.00 (−0.64 - 0.60) | 0.00 (−0.84 - 0.72) | 0.16 (−0.77 - 0.52) | 0.58 (−0.17 - 0.58) | 0.05 (−0.51 - 0.69) | −0.05 (−0.67 - 0.58) |
