# Supplementary material for: Predicting Prefecture-Level Well-Being Indicators in Japan Using Search Volumes in Internet Search Engines: Infodemiology Study
Source: J Med Internet Res. 2024 Nov 11;26:e64555. doi: 10.2196/64555 (PMC11589491; doi:10.2196/64555)
Supplement: Multimedia Appendix 5 [file jmir_v26i1e64555_app5.docx]

**Multimedia Appendix 5. Scores of the Regional Well−Being Index for the Year 2019**

| **Prefecture** | **Income** | **Jobs** | **Housing** | **Health** | **Work−Life Balance** | **Education** | **Community** | **Civic Engagement** | **Environment** | **Safety** | **Life Satisfaction** |
| --- | --- | --- | --- | --- | --- | --- | --- | --- | --- | --- | --- |
| Hokkaido | −0.38 | −1.28 | −1.07 | −1.74 | 0.21 | −0.67 | −0.22 | 1.15 | 0.33 | 0.14 | −0.93 |
| Aomori | −0.79 | −1.03 | 0.67 | −1.52 | −1.50 | 0.19 | −0.65 | −1.37 | 0.33 | −0.20 | −0.57 |
| Iwate | −0.48 | −0.03 | 0.82 | −2.09 | −1.69 | −1.02 | −2.37 | 1.81 | 0.33 | −0.57 | −2.05 |
| Miyagi | −0.15 | −1.28 | −0.68 | 0.44 | −0.47 | −0.50 | −1.00 | 0.55 | 0.33 | 0.54 | −0.26 |
| Akita | −0.62 | −1.28 | 1.26 | −0.05 | −1.31 | 2.09 | −1.05 | 1.74 | 0.33 | 0.56 | −1.49 |
| Yamagata | −0.22 | 0.96 | 1.38 | 0.02 | −1.23 | −0.67 | −0.01 | 2.78 | 0.33 | 0.75 | −2.05 |
| Fukushima | −0.15 | −0.03 | 0.43 | −0.60 | −1.09 | −1.02 | −0.67 | 0.84 | 0.33 | 1.48 | −0.65 |
| Ibaraki | 0.47 | −0.78 | 0.06 | 0.12 | 0.11 | 0.54 | 0.13 | −0.89 | 0.33 | −0.38 | 0.58 |
| Tochigi | 0.68 | −0.28 | −0.05 | −0.03 | 0.01 | −0.15 | −0.68 | −1.09 | 0.33 | −0.79 | 0.15 |
| Gunma | 0.55 | −0.28 | −0.06 | 1.29 | −0.49 | 0.36 | −1.26 | −0.15 | 0.33 | −0.01 | 0.51 |
| Saitama | 0.04 | −0.53 | −1.05 | 1.41 | 2.01 | 0.02 | −0.19 | −0.55 | 0.33 | −1.49 | 0.54 |
| Chiba | 0.08 | −0.03 | −1.02 | −0.05 | 1.45 | −0.33 | −1.24 | −0.83 | 0.33 | −0.32 | 0.15 |
| Tokyo | 5.57 | −0.78 | −2.77 | 0.51 | 0.81 | 1.23 | −1.30 | 0.69 | 0.33 | −0.40 | 0.92 |
| Kanagawa | 0.37 | −0.03 | −1.73 | 0.86 | 1.68 | 0.54 | −0.21 | −0.02 | 0.33 | 0.41 | 0.96 |
| Niigata | −0.13 | −0.03 | 1.11 | −0.05 | 0.13 | 0.19 | −0.05 | 1.51 | 0.33 | −0.04 | 0.01 |
| Toyama | 0.61 | 0.96 | 1.93 | 0.12 | −0.51 | 1.74 | −1.26 | −0.45 | 0.33 | −1.47 | −2.20 |
| Ishikawa | −0.09 | 1.21 | 0.82 | 0.74 | −0.45 | 2.09 | −1.05 | −0.43 | 0.33 | 1.83 | 0.51 |
| Fukui | 0.63 | 1.71 | 1.59 | 0.94 | −1.25 | 2.43 | 0.63 | −0.28 | 0.33 | −1.89 | −0.53 |
| Yamanashi | 0.22 | 0.22 | 0.23 | 1.56 | −0.05 | 0.36 | 0.12 | 0.64 | 0.33 | 0.65 | 1.50 |
| Nagano | −0.19 | 0.22 | 0.72 | −0.15 | 0.03 | −0.15 | −0.04 | 1.28 | 0.33 | 0.49 | 0.08 |
| Gifu | 0.04 | 1.96 | 1.37 | 0.74 | −0.12 | 0.88 | 0.22 | 0.51 | 0.33 | −0.15 | −0.24 |
| Shizuoka | 0.79 | 0.22 | −0.17 | 1.36 | −0.03 | 1.23 | 0.11 | 0.38 | 0.33 | −1.00 | 0.59 |
| Aichi | 1.31 | 0.72 | −0.61 | 0.35 | 0.26 | 0.71 | 0.33 | −0.15 | 0.33 | −0.69 | 1.56 |
| Mie | −0.06 | 1.96 | 0.65 | 0.44 | 0.32 | 0.02 | 0.88 | 0.67 | 0.33 | 1.23 | 0.86 |
| Shiga | 0.62 | 0.72 | 0.97 | 1.38 | 0.65 | −1.02 | 0.99 | 0.73 | 0.33 | 0.11 | 0.33 |
| Kyoto | −0.02 | −0.78 | −0.91 | 0.12 | 2.26 | 0.36 | −1.47 | −0.56 | 0.33 | 1.04 | −1.09 |
| Osaka | 0.08 | −2.02 | −1.59 | −1.27 | 1.14 | −0.67 | −0.58 | −0.05 | 0.33 | −3.15 | −0.17 |
| Hyogo | 0.04 | −0.53 | −0.52 | −0.26 | 1.58 | 0.88 | 1.25 | −0.05 | 0.33 | −0.52 | 0.11 |
| Nara | −0.59 | 0.47 | 0.75 | 0.10 | 2.84 | −0.15 | −0.14 | 0.17 | 0.33 | 0.80 | −0.01 |
| Wakayama | −0.06 | 1.21 | 0.34 | −0.42 | 0.73 | −0.67 | 0.72 | 0.37 | 0.33 | 0.07 | 0.13 |
| Tottori | −1.18 | −0.53 | 1.03 | −1.77 | −0.55 | −0.15 | −0.73 | 0.27 | 0.33 | −0.20 | −0.94 |
| Shimane | −0.13 | 1.21 | 1.04 | −0.08 | −0.94 | −0.84 | −1.27 | 1.22 | 0.33 | 0.27 | −1.35 |
| Okayama | −0.45 | −0.78 | 0.23 | −0.60 | −0.05 | −0.15 | 1.48 | −0.87 | −4.78 | −0.08 | 1.43 |
| Hiroshima | 0.28 | −0.78 | −0.52 | 0.12 | −0.40 | 0.36 | 0.44 | −0.97 | −1.23 | 0.49 | −0.15 |
| Yamaguchi | 0.47 | 0.72 | 0.02 | 1.13 | 0.01 | 0.36 | 1.35 | −0.35 | 0.33 | −0.83 | −0.07 |
| Tokushima | 0.28 | 0.47 | 0.46 | −0.85 | −0.69 | −0.33 | 0.34 | −2.39 | 0.33 | 0.94 | −0.32 |
| Kagawa | 0.01 | 0.22 | 0.53 | −0.50 | −0.32 | 0.02 | −1.26 | −0.82 | 0.33 | −0.25 | −0.96 |
| Ehime | −0.61 | 0.96 | −0.10 | −1.91 | 0.19 | 0.54 | 1.95 | 0.83 | 0.33 | 1.37 | −0.87 |
| Kochi | −0.72 | 0.47 | −0.22 | −1.69 | 0.22 | −1.19 | 1.08 | −0.58 | 0.33 | −1.27 | −0.02 |
| Fukuoka | −0.36 | −1.78 | −1.36 | −0.70 | 0.67 | −0.50 | 0.23 | −1.39 | −0.55 | −0.21 | 0.22 |
| Saga | −0.33 | 0.47 | 0.45 | 0.51 | −1.50 | −1.53 | 1.27 | −0.84 | −2.11 | −0.27 | 0.53 |
| Nagasaki | −0.74 | −0.28 | −0.35 | −0.58 | −0.84 | −0.15 | 1.88 | −0.78 | 0.33 | 1.07 | −0.39 |
| Kumamoto | −0.62 | −1.53 | −0.45 | −0.67 | −0.36 | −0.84 | 0.25 | −0.37 | −3.20 | 1.20 | −0.48 |
| Oita | −0.65 | 0.22 | −0.17 | 1.81 | −0.49 | 0.36 | 1.82 | 0.40 | −1.49 | 0.17 | 0.93 |
| Miyazaki | −1.20 | 1.71 | −0.69 | 1.11 | −0.34 | −0.50 | 0.05 | −1.64 | 0.33 | 1.79 | 1.91 |
| Kagoshima | −0.93 | −0.78 | −0.96 | 1.28 | −0.51 | −1.36 | 0.57 | −0.72 | 0.33 | 0.38 | 0.89 |
| Okinawa | −1.26 | −1.53 | −1.82 | −0.88 | −0.12 | −2.91 | 0.60 | 0.04 | 0.33 | −1.61 | 2.40 |
| Median (IQR) | −0.09 (−0.53 - 0.28) | −0.03 (−0.78 - 0.72) | 0.02 (−0.64 - 0.74) | 0.02 (−0.60 - 0.74) | −0.05 (−0.51 - 0.29) | −0.15 (−0.67 - 0.45) | 0.05 (−0.71 - 0.61) | −0.05 (−0.75 - 0.65) | 0.33 (0.33 - 0.33) | −0.01 (−0.46 - 0.60) | 0.01 (−0.55 - 0.56) |
