# Supplementary material for: Predicting Prefecture-Level Well-Being Indicators in Japan Using Search Volumes in Internet Search Engines: Infodemiology Study
Source: J Med Internet Res. 2024 Nov 11;26:e64555. doi: 10.2196/64555 (PMC11589491; doi:10.2196/64555)
Supplement: Multimedia Appendix 6 [file jmir_v26i1e64555_app6.docx]

**Multimedia Appendix 6. Representative Words in the Better Life Index Domains and Related Words for Each Domain.**

**Table S1. Income**

| **Japanese words** | **English words** | **Income of RWI**^d^ | **Integrated RWI**^e^ |
| --- | --- | --- | --- |
| 所得^a^ | income | −0.0652 | −0.1130 |
| 所得 控除 | income deduction | 0.0248 | −0.0128 |
| 控除 | deduction | 0.0085 | 0.0847 |
| 給与 所得 | employment income | 0.0585 | −0.0532 |
| 給与^b^ | salary | −0.0172 | −0.1441 |
| 所得 申告^b^ | income declaration | −0.0661 | −0.0660 |
| 所得 証明^b^ | income certificate | −0.3012 | −0.2802 |
| 給与 所得 控除 | employment income deduction | −0.0554 | −0.0117 |
| 所得 証明 書 | income certificate | −0.2508 | −0.2102 |
| 給与 所得 者 | salaried employee | −0.0173 | 0.0345 |
| 確定 申告^b^ | tax return | −0.0327 | 0.2642 |
| 扶養 所得^b^ | dependent income | −0.1692 | −0.0419 |
| 課税 所得 | taxable income | −0.0097 | 0.0107 |
| 年金 | pension | −0.1556 | −0.0062 |
| 所得 計算 | income calculation | −0.1155 | −0.0191 |
| 年金 所得 | pension income | 0.0023 | −0.0435 |
| 源泉 所得 | source of income | 0.0033 | −0.0062 |
| 譲渡 所得^b^ | capital gains | 0.0259 | 0.1116 |
| 所得 金額 | income amount | 0.0103 | 0.0446 |
| 退職 所得^b^ | retirement income | 0.2078 | 0.2080 |
| 所得税 | income tax | 0.0903 | −0.0485 |
| 源泉 徴収 | withholding tax | 0.0248 | −0.0396 |
| 所得 収入 | taxable income income | −0.0976 | −0.0814 |
| 扶養 控除 | dependent deduction | 0.0308 | 0.1024 |
| 住民 税^b^ | resident tax | 0.2263 | 0.1650 |
| 所得 制限 | income cap | −0.0774 | −0.0128 |

**Table S2. Jobs**

| **Japanese words** | **English words** | **Jobs of RWI^d^** | **Integrated RWI^e^** |
| --- | --- | --- | --- |
| 雇用^a^ | employment | −0.0560 | −0.1532 |
| 雇用 保険 | employment insurance | −0.2162 | −0.2871 |
| 障害 者^c^ | handicapped | - | - |
| 雇用 保険 被 保険 者 | employment insurance insured person | −0.1434 | −0.1421 |
| 障害 者 雇用 | employment of the handicapped | 0.1327 | 0.0960 |
| 雇用 契約^b^ | employment contract | −0.0277 | 0.0908 |
| 雇用 保険 証^b^ | employment insurance certificate | −0.1191 | 0.0092 |
| 雇用 保険 者 証^b^ | employment insurance certificate | −0.1168 | −0.1827 |
| 雇用 保険 資格 | Employment Insurance Eligibility | −0.2600 | −0.0616 |
| 雇用 保険 被 保険 者 証^b^ | employment insurance certificate | −0.0257 | 0.0139 |
| 雇用 保険 料 | employment insurance premium | −0.1156 | −0.0899 |
| 雇用 保険 と は | What is employment insurance? | −0.2328 | −0.2065 |
| 雇用 保険 加入 | employment insurance enrollment | −0.0790 | 0.0980 |
| 助成 金 雇用^b^ | subsidy employment | 0.0172 | −0.0897 |
| ハロ−ワ−ク | hello work (Public Employment Security Office) | −0.0485 | −0.1459 |
| 年金^c^ | pension | - | - |
| 雇用 保険 料率^b^ | employment insurance premium rate | −0.0291 | −0.0821 |
| 雇用 契約 書 | employment agreement | −0.2242 | −0.1057 |
| 雇用 条件 | employment conditions | −0.2499 | 0.0133 |
| 社会 保険 | social insurance | −0.1783 | −0.2047 |
| 雇用 保険 社会 保険 | employment Insurance Social Insurance | −0.1226 | −0.0304 |
| 失業 保険^b^ | unemployment insurance | −0.2202 | −0.4092 |
| 雇用 統計 | employment statistics | 0.0881 | 0.0815 |
| 雇用 保険 失業 保険 | employment insurance unemployment insurance | −0.3147 | −0.1385 |
| ハロ−ワ−ク 雇用 保険 | hello work employment insurance | −0.2842 | −0.0983 |
| 派遣 | Temporary staffing | 0.1086 | 0.1175 |

**Table S3. Housing**

| **Japanese words** | **English words** | **Housing of RWI^d^** | **Integrated RWI^e^** |
| --- | --- | --- | --- |
| 住宅^a,b^ | housing | −0.2152 | −0.2834 |
| 住宅 ロ−ン | housing loan | −0.1304 | 0.0196 |
| 中古 住宅 | used housing | 0.0962 | −0.0361 |
| 住宅 控除^b^ | housing deduction | 0.0742 | 0.2565 |
| 賃貸 住宅^b^ | rental housing | −0.4485 | −0.2929 |
| 金利 | interest rate | −0.0509 | 0.0602 |
| 市営 住宅^b^ | municipal house | −0.2833 | −0.3789 |
| 住宅 ロ−ン 金利 | housing loan interest rate | −0.1481 | 0.0647 |
| 住宅 ロ−ン 控除^b^ | housing loan deduction | 0.1003 | 0.1087 |
| 注文 住宅^b^ | custom housing | 0.0160 | 0.1404 |
| マンション^b^ | condominium | −0.6042 | −0.3894 |
| 住宅 展示 場^b^ | house exhibition place | −0.0529 | −0.0599 |
| 住宅 情報^b^ | house information | −0.3036 | −0.2874 |
| 住宅 保険 | home insurance | −0.1614 | −0.0486 |
| 高齢 者 住宅^b^ | elderly housing | −0.1468 | −0.1279 |
| 住宅 ロ−ン 審査 | housing loan screening | −0.2191 | −0.0426 |
| 県営 住宅 | prefectural housing | −0.2343 | −0.0980 |
| 住宅 地図^c^ | housing map | - | - |
| リフォ−ム^b^ | reform | 0.1606 | 0.0949 |
| 日本 住宅^c^ | japan housing | - | - |
| 住宅 ロ−ン 借り換え | housing loan refinancing | 0.0411 | −0.0023 |
| 住宅 購入 | housing purchase | −0.1108 | −0.0002 |
| 分譲 住宅^b^ | dwelling for sale | 0.1357 | 0.2038 |
| 住宅 メ−カ− | housing manufacturer | 0.1292 | 0.1487 |
| 住宅 ロ−ン 減税 | housing loan tax reduction | 0.1067 | 0.1087 |
| エコ ポイント 住宅^b^ | eco point housing (energy−efficient housing that meets government standards) | 0.2199 | 0.1727 |

**Table S4. Health**

| **Japanese words** | **English words** | **Health of RWI^d^** | **Integrated RWI^e^** |
| --- | --- | --- | --- |
| 健康^a^ | health | 0.1752 | 0.1923 |
| 健康 保険 | health insurance | −0.0551 | 0.0271 |
| 健康 診断 | health examination | 0.0564 | −0.0378 |
| 国民 健康 保険 | national health insurance | 0.0184 | 0.0029 |
| 健康 保険 組合^b^ | health insurance association | 0.2972 | 0.3618 |
| 健康 センタ−^b^ | health center | 0.2780 | 0.2280 |
| 健康 ランド^b^ | health land (Super Sento, Spa Resort) | 0.2187 | 0.3275 |
| 健康 保険 料 | health insurance premium | −0.0806 | 0.0006 |
| 健康 管理^b^ | health care | 0.1598 | 0.1089 |
| 健康 保険 協会^b^ | health insurance association | −0.1866 | −0.2088 |
| 国民 健康 保険 保険 料 | national health insurance premium | 0.0391 | 0.0026 |
| 健康 食品 | health food | 0.1559 | −0.0697 |
| 保険 証^b^ | insurance certificate | −0.0773 | −0.2121 |
| 国民 健康 保険 料 | national health insurance premium | 0.0846 | 0.0332 |
| 健康 保険 証 | health insurance certificate | 0.0049 | −0.0132 |
| 扶養^c^ | dependent (family member) | - | - |
| 健康 保険 扶養 | health insurance dependent | −0.1852 | −0.0581 |
| 社会 保険 | social insurance | −0.0845 | −0.2023 |
| 全国 健康 保険 協会^b^ | national health insurance association | −0.1102 | −0.2048 |
| 国民 年金 | national pension | −0.1160 | −0.0259 |
| 健康 保険 と は^b^ | What is health insurance? | −0.0456 | −0.1307 |
| 医療 保険 | medical insurance | −0.1477 | −0.1863 |
| 厚生 年金 | welfare pension | −0.1572 | 0.0330 |
| 健康 管理 センタ−^b^ | health management center | 0.2568 | 0.2128 |
| 退職 健康 保険 | retirement health insurance | −0.1229 | −0.0341 |
| 社会 保険 国民 健康 保険 | social insurance national health insurance | 0.2819 | 0.0299 |

**Table S5. Work-Life Balance**

| **Japanese words** | **English words** | **Work−Life Balance of RWI^d^** | **Integrated RWI^e^** |
| --- | --- | --- | --- |
| 残業^a^ | overtime | −0.1403 | 0.1117 |
| 残業 時間 | overtime hours | −0.0441 | 0.0794 |
| 残業 計算 | overtime calculation | 0.0295 | −0.0973 |
| 残業 手当^b^ | overtime pay | 0.0067 | −0.1559 |
| サ−ビス 残業 | overtime work without pay | 0.0786 | −0.0461 |
| 労働 時間^b^ | working time | −0.2853 | −0.1108 |
| みなし 残業 | fixed overtime | 0.0557 | −0.0172 |
| 仕事^c^ | work | - | - |
| 仕事 残業 | work overtime | 0.0967 | −0.0309 |
| 残業 代 計算 | overtime pay calculation | 0.1459 | −0.0109 |
| 労働 基準 法^b^ | labor standards act | −0.1064 | 0.0341 |
| 労働 基準 法 残業 | labor standards law overtime | 0.1350 | 0.1648 |
| 残業 なし | no overtime | 0.2354 | −0.0840 |
| 残業 平均 | overtime average | 0.2065 | −0.0855 |
| 深夜 残業 | late night overtime | 0.1068 | −0.1139 |
| 転職^b^ | job change | 0.3502 | 0.2577 |
| 固定 残業 | fixed overtime | 0.0944 | −0.0942 |
| 残業 上限 | overtime upper limit | 0.1055 | −0.0393 |
| 残業 代 請求 | request overtime pay | −0.1452 | 0.0261 |
| 36 協定 残業 | 36 agreement overtime | 0.0207 | −0.0847 |
| 月 残業 時間^b^ | month overtime hours | 0.1540 | −0.1651 |
| 36 協定 | 36 Agreement (the Labor Standards Act Article 36 Agreement on Specific Overtime Work, Holiday Work, and Scheduled Holidays) | −0.1935 | 0.0396 |
| 残業 割増 | overtime premium | 0.0171 | −0.1035 |
| 残業 時間 計算 | overtime hours calculation | 0.0492 | −0.1000 |
| 残業 代 未払い | overtime pay unpaid | −0.1499 | −0.0867 |
| 残業 英語^c^ | overtime english | - | - |

**Table S6. Education**

| **Japanese words** | **English words** | **Education of RWI^d^** | **Integrated RWI^e^** |
| --- | --- | --- | --- |
| 教育^a,b^ | education | −0.1367 | −0.2159 |
| 教育 大学^b^ | university of education | 0.0174 | −0.1130 |
| 教育 委員 会^b^ | educational committee | −0.3964 | −0.3216 |
| 教育 センタ− | education center | −0.0453 | −0.1157 |
| 教育 学部 | department of education | −0.0663 | 0.0037 |
| 通信 教育 | correspondence education | −0.1086 | −0.0408 |
| 特別 教育^b^ | special education | −0.1182 | −0.2302 |
| 英語 教育^c^ | english education | - | - |
| 中等 教育 学校 | secondary school | 0.0757 | 0.0418 |
| 大阪教育大学^c^ | Osaka kyoiku university | - | - |
| 教育 指導^b^ | education guidance | −0.0873 | −0.1757 |
| 教育 実習 | teaching practice | −0.1731 | −0.0205 |
| 教育 ロ−ン^b^ | education loan | −0.0747 | −0.2239 |
| 幼児 教育 | infant education | −0.0442 | 0.0144 |
| 教育 研究 所^b^ | education research institute | 0.0734 | 0.1388 |
| 特別 支援 教育 | special needs education | −0.1546 | −0.1146 |
| 総合 教育 センタ− | general education center | 0.0151 | −0.0116 |
| 愛知 教育 大学^c^ | Aichi university of education | - | - |
| 義務 教育^b^ | compulsory education | −0.0099 | 0.1680 |
| 京都 教育 大学^c^ | Kyoto university of education | - | - |
| 学校 教育 法 | school education Act | −0.1792 | −0.0278 |
| 教育 問題^b^ | educational problem | 0.2689 | 0.2153 |
| 通信 教育 大学 | correspondence college | −0.0020 | 0.0818 |
| 北海道 教育 大学^c^ | Hokkaido university of education | - | - |
| 東京 都 教育 委員 会^c^ | Tokyo metropolitan board of education | - | - |
| 教育 出版^c^ | education publication | - | - |

**Table S7. Community**

| **Japanese words** | **English words** | **Community of RWI^d^** | **Integrated RWI^e^** |
| --- | --- | --- | --- |
| 人間関係^a^ | human relationship | −0.1843 | 0.0196 |
| 人間 関係^c^ | human relationship | - | - |
| 職場^c^ | workplace | - | - |
| 人間 関係 職場 | human relationships workplace | −0.1361 | −0.0900 |
| 仕事 人間 関係 | work human relationship | −0.1449 | −0.0713 |
| 仕事^c^ | work | - | - |
| 職場 の 人間 関係 | human relationships at work | 0.0284 | −0.0798 |
| 会社^c^ | company | - | - |
| 人間 関係 と は | What is human relationship? | −0.1135 | −0.0599 |
| ストレス^c^ | stress | - | - |
| 人間 関係 ストレス | human relationship stress | −0.0013 | −0.0556 |
| 疲れ た^b^ | tired | −0.2156 | 0.0483 |
| 仕事 の 人間 関係 | human relationships at work | −0.0251 | −0.0471 |
| 人間 関係 悩み^b^ | human relationship worries | −0.0408 | −0.0513 |
| 人間 関係 占い | human relationship fortune telling | 0.0097 | −0.0643 |
| 大学 人間 関係^b^ | university human relationship | −0.0108 | 0.1483 |
| 友達^b^ | friend | 0.0633 | −0.0743 |
| 人間 関係 転職 | human relations career change | 0.0343 | 0.0229 |
| 渋谷 人間 関係^c^ | Shibuya human relationship | - | - |
| バイト 人間 関係 | part−time job human relationship | 0.0293 | −0.1441 |
| 人間 関係 うまくいかない | human relationships don't go well | −0.1076 | −0.0129 |
| 人間 関係 英語^c^ | human relationships english | - | - |
| 人間 関係 苦手^b^ | not good at human relationships | −0.0135 | 0.1638 |
| 人間 関係 名言 | human relationship quote | 0.0331 | −0.0213 |
| 名言^c^ | quote | - | - |
| 仕事 辞め たい | i want to quit my job | −0.0399 | −0.1646 |

**Table S8. Civic Engagement**

| **Japanese words** | **English words** | **Civic Engagement of RWI^d^** | **Integrated RWI^e^** |
| --- | --- | --- | --- |
| 政治^a,b^ | politics | −0.1161 | −0.0351 |
| 政治 経済 | politics economy | −0.0864 | 0.0597 |
| 政治 ブログ^b^ | politics blog | −0.0817 | −0.3548 |
| 日本 政治 | japan politics | −0.1040 | 0.1586 |
| ニュ−ス 政治 | news politics | −0.1073 | −0.0836 |
| 日本 の 政治 | japanese politics | −0.0911 | 0.0166 |
| 政治 ランキング^c^ | politics ranking | - | - |
| 国際 政治 | international politics | −0.2325 | −0.0711 |
| 政治 資金 | politics funds | −0.1920 | −0.1499 |
| 選挙^b^ | election | 0.2690 | 0.0999 |
| 政治 経済 学部^c^ | department of politics and economics | - | - |
| ブログ ランキング 政治^c^ | blog ranking politics | - | - |
| 政治 問題^b^ | political problem | −0.1939 | −0.0821 |
| 政治 英語^c^ | politics english | - | - |
| 韓国 政治^c^ | korea politics | - | - |
| 政治 意味 | politics meaning | −0.0291 | 0.0336 |
| 中国 政治^c^ | china politics | - | - |
| 政治 団体 | political organization | 0.0017 | −0.1019 |
| 政治 活動 | political activities | −0.1364 | −0.0679 |
| 早稲田 政治 経済^c^ | Waseda Politics Economy | - | - |
| 政治 まとめ | politics summary | 0.0426 | −0.0109 |
| 政治 速報 | politics breaking news | 0.0900 | −0.0509 |
| 政治 思想 | politics ideology | −0.0706 | −0.0467 |
| アメリカ 政治^c^ | America politics | - | - |
| 早稲田 大学^c^ | Waseda university | - | - |
| 人気 ブログ 政治^c^ | popular blog politics | - | - |

**Table S9. Environment**

| **Japanese words** | **English words** | **Environment of RWId** | **Integrated RWIe** |
| --- | --- | --- | --- |
| 環境^a^ | environment | 0.1037 | 0.0213 |
| 環境 センタ− | environmental center | −0.0069 | −0.0373 |
| 環境 変数^c^ | environment variables | - | - |
| 環境 開発^c^ | environment development | - | - |
| 日本 環境 | japan environment | 0.1747 | 0.0337 |
| 環境 問題 | environment problem | −0.0689 | 0.0405 |
| 環境 保全 | environmental conservation | 0.1823 | 0.0294 |
| 環境 研究 所 | environmental research institute | 0.2593 | 0.0417 |
| 環境 設定^c^ | environment setting | - | - |
| 地球 環境 | earth environment | 0.1181 | 0.1093 |
| 環境 整備^c^ | environmental improvement | - | - |
| 環境 生活 | environment life | 0.1105 | 0.0939 |
| 環境 基準^b^ | environmental standard | 0.0862 | −0.1589 |
| 自然 環境^b^ | natural environment | 0.2614 | 0.1366 |
| 環境 英語^c^ | environment english | - | - |
| ネット 環境^c^ | internet environment | - | - |
| 住 環境 コ−ディネ−タ−^c^ | housing environment coordinator | - | - |
| 福祉 住 環境^c^ | welfare housing environment | - | - |
| 作業 環境^c^ | work environment | - | - |
| 住 環境 福祉 コ−ディネ−タ−^c^ | housing environment welfare coordinator | - | - |
| 環境 教育 | environment education | 0.0757 | 0.0086 |
| 環境 汚染 | environmental pollution | 0.0911 | 0.0397 |
| 労働 環境^c^ | labor environment | - | - |
| 遊戯王 環境^c^ | Yu−Gi−Oh! environment | - | - |
| 家庭 環境^c^ | home environment | - | - |
| 環境 保護 | environmental protection | 0.0821 | 0.0043 |

**Table S10. Safety**

| **Japanese words** | **English words** | **Safety of RWI^d^** | **Integrated RWI^e^** |
| --- | --- | --- | --- |
| 治安^a,b^ | public safety | −0.1203 | −0.0417 |
| 治安 悪い | public safety unsafe | 0.0094 | −0.0832 |
| 大阪 治安^c^ | Osaka public safety | - | - |
| 治安 ランキング | public safety ranking | −0.0521 | 0.0257 |
| 日本 治安^b^ | Japan public safety | −0.1831 | −0.1191 |
| 東京 治安^c^ | Tokyo public safety | - | - |
| 世界 治安^c^ | world public safety | - | - |
| メキシコ^c^ | Mexico | - | - |
| メキシコ 治安^c^ | Mexico public safety | - | - |
| 海外 治安^c^ | overseas public safety | - | - |
| 治安 が 悪い | bad public safety | −0.1719 | −0.1507 |
| 京都 治安^c^ | Kyoto public safety | - | - |
| 川崎 治安^c^ | Kawasaki public safety | - | - |
| 福岡 治安^c^ | Fukuoka public safety | - | - |
| アメリカ 治安^c^ | America security | - | - |
| 治安 維持 法^c^ | public order and police law | - | - |
| アメリカ^c^ | America | - | - |
| タイ^c^ | Thailand | - | - |
| 治安 英語^c^ | public safety english | - | - |
| 足立 区 治安^c^ | Adachi ward public safety | - | - |
| 足立 区^c^ | Adachi ward | - | - |
| タイ 治安^c^ | Thailand public safety | - | - |
| 西成^c^ | Nishinari | - | - |
| 西成 治安^c^ | Nishinari public safety | - | - |
| セブ 治安^c^ | Cebu public safety | - | - |
| 韓国 治安^c^ | Korea public safety | - | - |

**Table S11. Life Satisfaction**

| **Japanese words** | **English words** | **Life Satisfaction of RWId** | **Integrated RWIe** |
| --- | --- | --- | --- |
| 幸せ^a,b^ | happiness | −0.1109 | −0.1683 |
| パン ケ−キ^c^ | pancake | - | - |
| 幸せ の パン^c^ | bread of happiness | - | - |
| 幸せ パン ケ−キ^c^ | happiness pancake | - | - |
| 幸せ の パン ケ−キ^c^ | pancake of happiness | - | - |
| 幸せ の 時間^b^ | happy time | −0.0138 | −0.0960 |
| 幸せ に なる^b^ | be happy | 0.1150 | −0.0025 |
| 幸せ 歌詞^c^ | happiness lyrics | - | - |
| 幸せ 英語^c^ | happiness english | - | - |
| 結婚 幸せ | marriage happiness | −0.0765 | −0.0764 |
| 結婚 | marriage | 0.0088 | −0.0431 |
| 幸せ 意味 | happiness meaning | 0.1926 | −0.0286 |
| 幸せ に なれる | can be happy | −0.0424 | −0.0892 |
| 幸せ ます^c^ | Happiness (local dialect) | - | - |
| 幸せ 画像^b^ | happiness images | −0.0952 | −0.1730 |
| 幸せ に なりたい^b^ | want to be happy | 0.2190 | −0.1193 |
| 幸せ に な ろう^b^ | let's be happy | −0.0425 | −0.2178 |
| 幸せ の レシピ^c^ | recipe for happiness | - | - |
| しあわせ | happiness | −0.1071 | 0.0149 |
| 彼氏 幸せ | boyfriend happiness | −0.0696 | −0.1057 |
| 幸せ に な ろう よ | let's be happy | 0.1421 | 0.0987 |
| 幸せ に なる 方法 | how to be happy | −0.0062 | −0.0564 |
| 幸せ の 黄色い^c^ | happy yellow | - | - |
| 幸せ な 結婚 | a happy marriage | −0.1122 | 0.0017 |
| 幸せ に なる ため に^b^ | to be happy | 0.0071 | −0.1232 |
| 小さな 幸せ^b^ | small happiness | −0.0886 | −0.1966 |

a: representative words for each domain of the RWI.

b: Words selected for model created by Elastic net.

c: Words excluded as irrelevant to the corresponding domain.

d: Partial correlation coefficients with the corresponding domain of the Regional Well−being Index (RWI).

e: Partial correlation coefficients with the corresponding Integrated RWI.
